# Subversion of Atypical Mucin Traps by a Spore‐Coat Effector Blocks Cellular Immunity in Drosophila

**DOI:** 10.1002/advs.76623

**Published:** 2026-07-16

**Authors:** Shiqin Li, Haimin Chen, Gangqi Fang, Dongxiang Wei, Hongyun Wu, Chen Chen, Song Hong, Chengshu Wang

**Affiliations:** ^1^ Key Laboratory of Insect Developmental and Evolutionary Biology State Key Laboratory of Plant Trait Design CAS Center for Excellence in Molecular Plant Sciences Shanghai Institute of Plant Physiology and Ecology Chinese Academy of Sciences Shanghai China; ^2^ School of Life Science and Technology ShanghaiTech University Shanghai China; ^3^ CAS Center for Excellence in Biotic Interactions University of Chinese Academy of Sciences Beijing China

**Keywords:** cellular immunity, entrapment, metarhizium, mucin‐like protein, spore‐coat effector

## Abstract

While a few effectors of entomopathogenic fungi such as *Metarhizium robertsii* have been shown to evade insect humoral immunity, the strategies employed by fungi to subvert host cellular defenses remain elusive. Here, we report the identification of a spore‐coat protein Eac1 in *M. robertsii* that is essentially required for fungal infection of drosophilids but not caterpillars. Eac1 targets Sgf1 (spore gluing factor), which interacts with its clustered homolog Sgf2 in *Drosophila melanogaster*. Both Sgf1 and Sgf2 are drosophilid‐specific secreted proteins of previously unknown function. Unlike *Sgf2*, *Sgf1* is patchily distributed among drosophilids and appears to be a duplicate of *Sgf2*. Both genes are induced via the Toll pathway following fungal infection. We demonstrate that Sgf1 and Sgf2 are small atypical mucins that bind fungal cell wall components and entrap spores by forming a colloidal‐like gel matrix; however, this entrapment can be disrupted by Eac1. Null mutants of *Sgf1*, *Sgf2*, and especially the double mutants of *Drosophila*, were significantly impaired in their ability to combat fungal colonization. Our findings reveal that spore entrapment by atypical mucins is a prerequisite for effective hemocyte encapsulation and demonstrate how fungal parasites deploy a specialized coat protein to evade host cellular immunity.

## Graphical abstract

While the entomopathogenic fungus *Metarhizium robertsii* can evade insect humoral immunity, its subversion of cellular defenses is still elusive. We identify Eac1 as a spore‐coat protein essential for infecting drosophilids by targeting atypical mucins (Sgf1 and Sgf2), which can entrap spores in a gel matrix and thereby facilitate hemocyte encapsulation. Remarkably, Eac1 disrupts this entrapment, allowing the fungus to evade host cellular immunity.

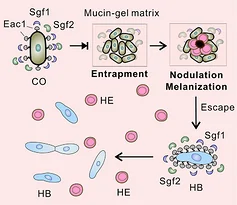

## 1 Introduction

Entomopathogenic fungi play an essential role in regulating insect populations, and different species have been developed as ecofriendly insect biocontrol agents [1]. Species like *Metarhizium robertsii* and *Beauveria bassiana* are being investigated as genetically tractable systems to elucidate the mechanistic interactions between fungi and insects. Diverse fungal effectors have been shown to function sequentially in evading host immunity [2]. For example, similar to plant pathogens [3], lysin motif effectors are required for the virulence of *B. bassiana* against insects by camouflaging the chitin oligomers to evade host immunity [4]. The metalloprotease MrM35‐4 secreted by *M. robertsii* cleaves insect prophenoloxidases into non‐functional fragments [5]. The small effectors Tge1 and Fkp1, secreted by *M. robertsii*, suppress the dual recognition machinery in *Drosophila melanogaster* by blocking GNBP3/GNBP‐like 3 binding/recognition and the CtsK1‐Psh protease cascade, respectively [6, 7]. A recent study has shown that the dual effectors ETS1 and ETS6 of *M. robertsii* target the Toll receptor ligand Spätzle of diverse insects for degradation and hijacking, respectively, to facilitate fungal infection [8]. More directly, the host‐preference effector Bhe1 enables *B. bassiana* to target a drosophilid‐specific factor Dsff1, and blocks its interaction with Bbd, thereby subverting the secretion of antimicrobial peptides (AMPs) in drosophilids [9]. To date, we have little evidence of virulence factors targeting cellular defense.

Cellular immune response in insects precedes humoral immunity [10, 11]. Our mechanistic understanding of cellular immune responses has historically lagged behind that of humoral immunity [12]. Relative to the well‐established antifungal Toll pathway for humoral immune responses, only a handful of genes have been characterized in the control of cellular immunity [11, 13, 14]. The hemocyte‐specific transmembrane protein Eater was first identified as an essential receptor for mediating the phagocytosis of bacteria [15, 16]. The Eater‐homologous receptor NimC1 plays a distinct role in microbial uptake, but they synergistically contribute to bacterial phagocytosis [17]. Similar to their functions in mammals, the immune‐inducible thioester‐containing proteins (TEPs) in insects participate in the cellular immune defense against fungi, bacteria, and parasitic wasp eggs [18]. The encapsulation/nodulation of large targets (e.g., fungal cells parasitoid eggs) in *Drosophila* is understood as a two‐step process: initial recognition and tagging by plasmatocytes, followed by multilayered capsule formation by lamellocytes [13]. Fungal infection, whether by natural means or intra‐hemocoel injection, results in the concurrent entry of many fungal cells into the hemocoel [19]. In contrast, a parasitoid attack typically involves only a single egg [20]. Our current understanding of encapsulation/nodulation cannot adequately explain why multiple fungal cells become entrapped within individual nodules [4].

Mucins, the gel‐forming constituents of mucus, play essential roles in trapping and immobilizing pathogens in humans [21, 22]. Like vertebrates, insects encode diverse glycosylated mucins. For example, 30 mucins and mucin‐related proteins have been identified in *D. melanogaster*. They are typically rich in proline, threonine, and serine (PTS) residues, vary in size and domain architectures, and contribute to development and physiology [23]. The *Drosophila* mucin Muc68D is an essential component of the intestinal peritrophic matrix to mediate barrier protection against bacterial infection [24]. Before this study, it was unclear whether insect mucins or mucin‐like proteins play a role in defense against fungal parasites.

Following our previous screening assays [6], we report in this study the identification of a putative glycosylphosphatidylinositol (GPI) cell‐surface anchoring protein, MAA_00324 (termed Eac1 for effector anti‐clumping), which is essential for *M. robertsii* virulence against flies. It subverts the function of previously unsuspected atypical mucin‐like proteins in *Drosophila*, thereby preventing spore entrapment and encapsulation by the host's cellular immunity.

## 2 Results

### 2.1 Spore Surface‐Localized Eac1 is a Virulence Factor Against *Drosophila*

Eac1 is a secreted protein rich in glycine (60/249 residues) and asparagine (32/249), and predicted to contain a C‐terminal GPI‐anchoring site (*p =* 1.97e‐4) (Figure 1A). Reverse transcription and quantitative PCR (RT‐qPCR) analysis revealed that *Eac1* expression was highest in the mature conidia of *M. robertsii*, followed by that in hyphal body cells harvested from the body cavity of wax moth larvae (*Galleria mellonella*) (Figure S1A). To confirm the protein localization feature, we performed a fusion PCR by inserting the green fluorescent protein (GFP) gene in frame after the signal peptide region of Eac1 (instead of its C‐terminal) to avoid any disruption of the GPI anchoring function. The cassette was made under the control of the *Eac1* promoter (Pro_Eac1_::*SP‐GFP‐Eac1*) and used to transform the wild‐type (WT) strain of *M. robertsii*. In contrast to the sole GFP‐only control strain, which showed a diffuse cytosolic fluorescence in all type cells (Figure S2A), Eac1‐GFP clearly localized to the surface of both conidial and hyphal‐body cells. Interestingly, the GFP signal was not observed on the newly formed appressorium cells but on the mother cells (Figure 1B). Consistent with its basal expression pattern, the GFP‐Eac1 signal was not detected on the surfaces of mycelia harvested from a saprophytic liquid medium (Figure S2B).

**FIGURE 1:**
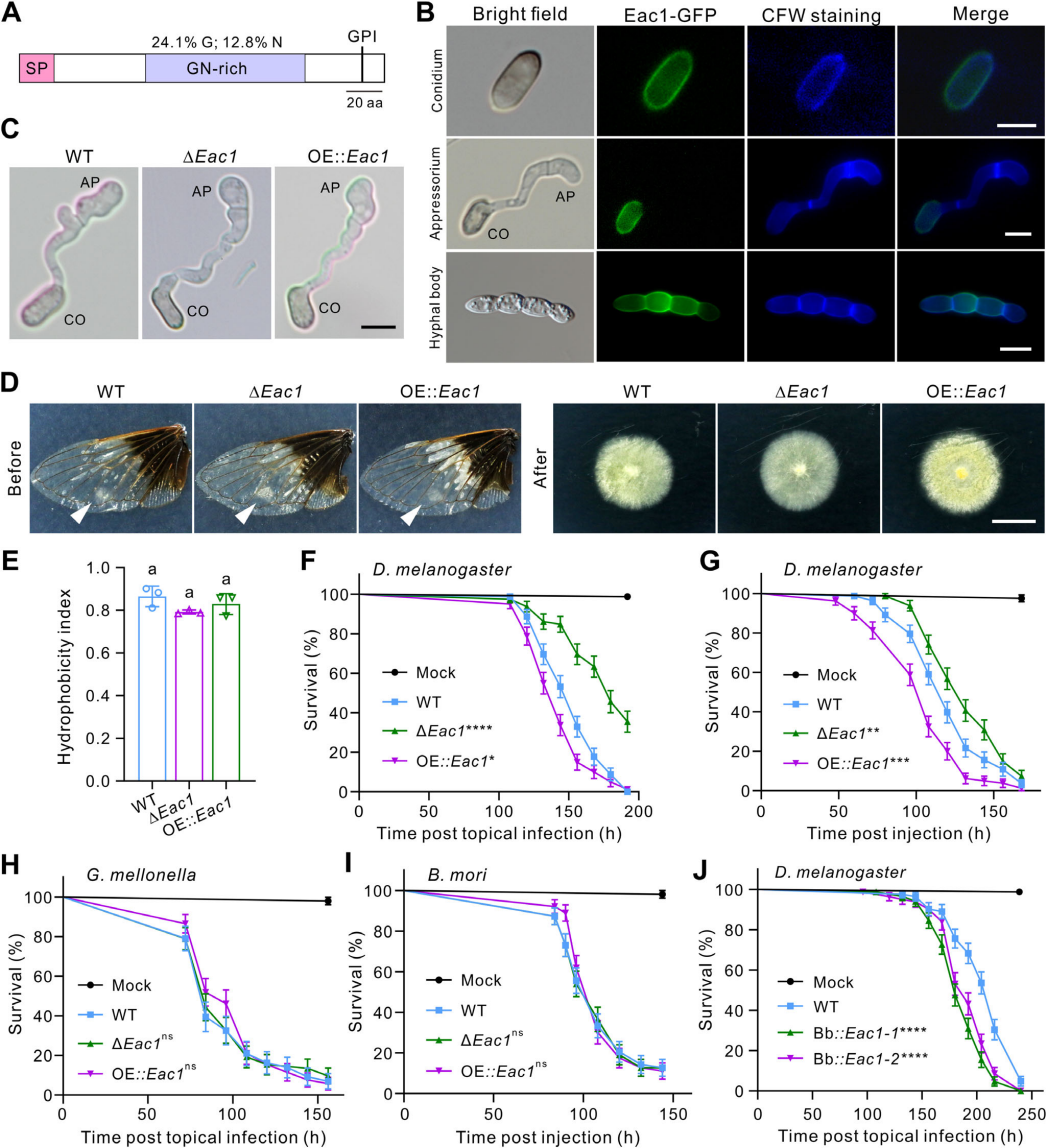
The spore surface Eac1 is required for fungal virulence. (A) Schematic structure of Eac1. SP, signal peptide; GN‐rich, glycine/asparagine‐rich; GPI, glycosylphosphatidyl‐inositol anchoring site. (B) Protein localization assays. CO, conidium; AP, appressorium; CFW, Calcofluor white. Appressorium was induced on a hydrophobic surface for 18 h, and hyphal‐body cells were harvested from the hemolymph of wax moth larvae after injection with the WT spores of *M. robertsii* for 36 h. Bar, 5 µm. (C) Deletion or overexpression of *Eac1* in *M. robertsii* did not impair appressorium differentiation. Bar, 5 µm. (D) Deletion or overexpression of *Eac1* in *M. robertsii* did not impair fungal penetration of cicada wings. Cicada wings were placed on 0.7% agar plates and individually inoculated with 2 µl of spore suspension (5 × 10^6^ conidia/mL; arrowed). After incubation for two days (left panels), the wings were removed and the plates were incubated for five additional days (right panels). Bar, 1 cm. (E) Deletion or overexpression of *Eac1* in *M. robertsii* did not affect conidial spore surface hydrophobicity. Conidia were harvested from two‐week old PDA plates. One‐way ANOVA followed by Tukey's test: the same letter above the bars, *p* > 0.05. (F,G) Differential survival of female flies after topical infection (F) and intra‐hemocoel injection (G) with the WT and *Eac1* mutants of *M. robertsii*. (H,I) Deletion of *Eac1* did not affect fungal virulence during topical infection of the wax moth (H) and silkworm (I) larvae. (J) Overexpression of *Eac1* in *B. bassiana* increases fungal virulence against female flies. Survival assays were conducted in a growth chamber at 25°C. Panels F‐J: plotted values are the mean ± SEM. Log‐rank test between WT and individual mutants: ^*^ *p <* 0.05; ^**^ *p* < 0.01; ^***^ *p* < 0.001; ^****^ *p <* 0.0001; ns, not significant.

We next performed deletion and overexpression of *Eac1* in *M. robertsii*. The obtained mutants were verified by PCR and RT‐qPCR (Figure S1B,C). Mutant isolates had no phenotypic difference from the WT strain when grown on potato dextrose agar (PDA) or PDA amended with different stress factors (Figure S1D). The WT and *Eac1* mutants showed no difference in appressorium differentiation (Figure 1C), and they thus had similar capacities to penetrate the cicada wings (Figure 1D). They also exhibited similar spore surface hydrophobicity (Figure 1E). Subsequent topical infection assays revealed that, however, the null mutant Δ*Eac1* was substantially impaired (Log‐rank test: *p* < 0.0001) in killing *D. melanogaster* females, while the virulence of the overexpression mutant OE::*Eac1* was substantially increased (*p* < 0.05) compared with the WT strain (Figure 1F). Similar survival patterns were observed after intra‐hemocoel injection of female flies with the WT and mutant strain spores (Figure 1G). The virulence of Δ*Eac1* was similarly reduced, whereas there was no statistical difference between the WT and OE::*Eac1* during topical infection of male flies (Figure S1E). Injecting male flies resulted in a similar survival pattern in females (Figure S1F). Intriguingly, both wax moth and silkworm (*Bombyx mori*) larvae were equally susceptible to topical infection by WT and *Eac1* mutants (Figure 1H,I).

Our genome survey indicated that *Eac1* is present exclusively in *Metarhizium* fungi and evolved in association with *Metarhizium* speciation (Figure S1G). To further verify its virulence contribution, we overexpressed *Eac1* in *B. bassiana* (Figure S1H). Subsequent survival assays confirmed that *Eac1* transformation significantly increased fungal virulence against *D. melanogaster* (Figure 1J). Collectively, we identified the *Metarhizium*‐specific *Eac1* that contributes to fungal infection of *Drosophila* but not caterpillars.

### 2.2 Eac1 Interacts With a *Drosophila* Factor Required for Antifungal Immunity

Next, based on the yeast two‐hybrid (Y2H) system protocol, we used Eac1 as bait to screen the yeast cDNA library that we generated from the RNA samples isolated from the immune‐challenged *D. melanogaster* [6, 25]. Positive clones were selected for sequencing, and 11 clones belonging to six *Drosophila* genes were obtained without frameshifts, including five clones identified as CG34054 (Table S1). Further Y2H analyses were conducted by cloning full‐length open reading frames (ORFs) of these genes for one‐by‐one verification of their interaction with Eac1. Positive interaction was only evident between Eac1 and CG34054 (Figure 2A). We checked and found that CG34054 (termed Sgf1: for spore gluing factor as indicated below) is a secreted protein of unknown function. Tag‐fused proteins, Eac1‐His and Sgf1‐GST (glutathione S‐transferase), were then expressed in *Escherichia coli* cells and purified (Figure S3A). Subsequent protein pull‐down analysis confirmed the positive interaction between Eac1 and Sgf1 (Figure 2B). We also co‐transfected Sf9 insect cells with the recombinant baculoviruses containing the *Sgf1*‐Myc and *Eac1*‐His fused genes for protein co‐expression and co‐immunoprecipitation (co‐IP) analysis. The results revealed that the Ni‐NTA (nickel‐nitrilotriacetic acid) resin successfully captured Eac1 along with Sgf1 (Figure 2C).

**FIGURE 2:**
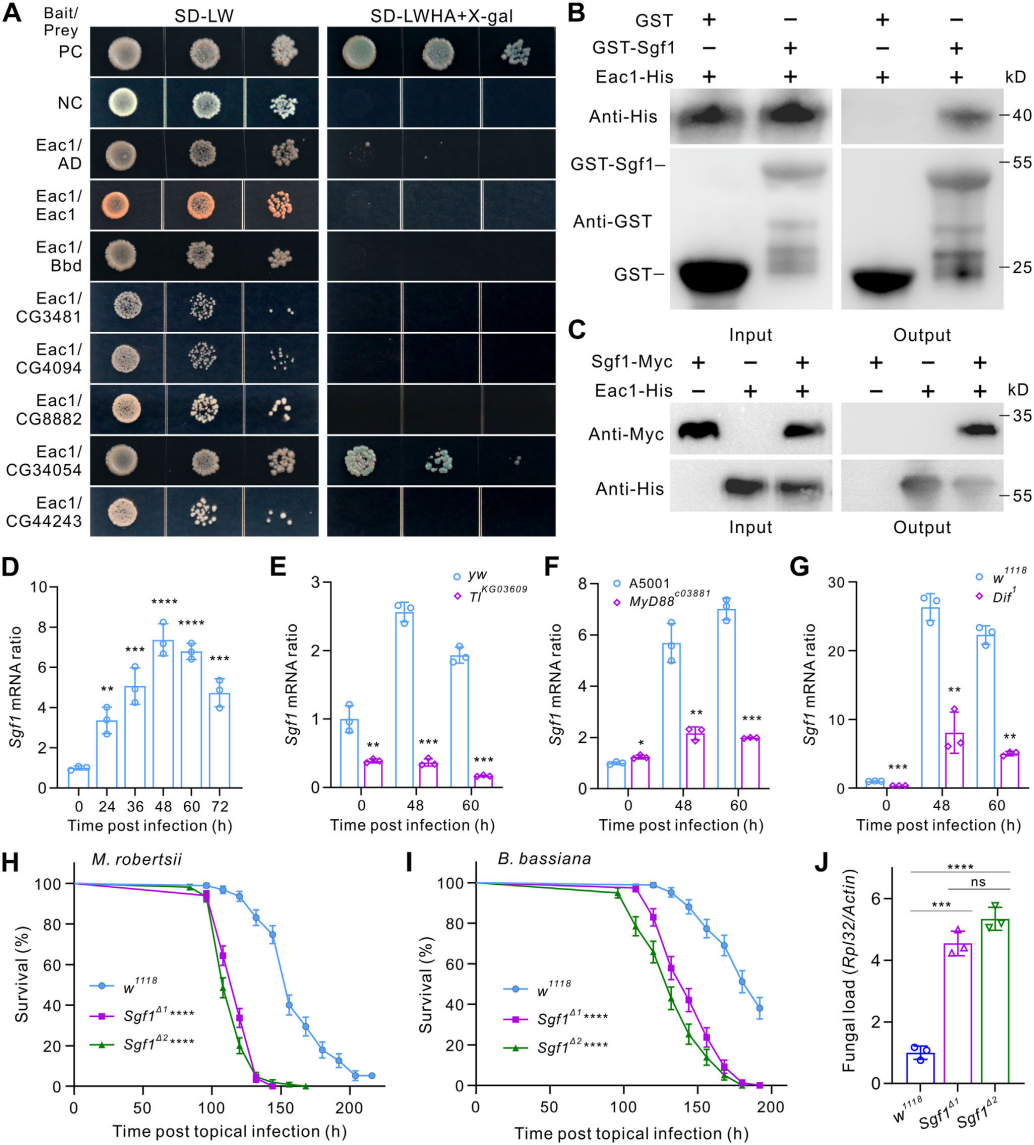
Eac1 interacts with a *Drosophila*‐specific factor that mediates antifungal defense. (A) Y2H verification of the library screen shows that Eac1 positively interacts with CG34054 (Sgf1). SD‐LW: synthetic dropout (SD) medium lacking Leu and Trp; SD‐LWHA: SD medium lacking Leu, Trp, His, and Ala. PC, positive control; NC, negative control. AD, blank pGADT7 vector. (B) Protein pull‐down analysis confirms the interaction between Eac1 and Sgf1. GST, glutathione S‐transferase; Myc, a peptide tag derived from the human c‐Myc protein. Original blots can be found in Figure S11A. (C) Immunoprecipitation (IP) analysis confirms the successful pull‐down of Sgf1‐Myc by Eac1‐His after co‐expression in Sf9 cells. Original blots can be found in Figure S11B. (D) Inductive expression of *Sgf1* in *D. melanogaster* after topical fungal infection for different durations. The significance of the difference was compared between the mock (0 h) and other times after treatment. (E–G) *Sgf1* is a downstream factor of the Toll pathway by showing significantly reduced expression in *Tl^KG03609^* (E), *MyD88^c03881^* (F), and *Dif^1^* (G) mutant *Drosophila* after topical challenge with the WT *Metarhizium* spores. (H,I) Topical infection assays show that deletion of *Sgf1* in *D. melanogaster* significantly impaired females’ antifungal defenses against *M. robertsii* (H) and *B. bassiana* (I). Two independent *Sgf1* mutants were included in bioassays. (J) Fungal load assay confirms that *Sgf1‐*null flies were significantly impaired in defending against colonization by WT *M. robertsii* after topical infection. Panels D‐G and J: Values are the mean ± SD. Pairwise two‐tailed Student's *t*‐test: ^**^ *p <* 0.01; ^***^ *p <* 0.001; ^****^ *p <* 0.0001; ns, not significant. Panels H and I: Plotted values are the mean ± SEM. Log‐rank test: ^****^ *p <* 0.0001.

To determine whether *Sgf1* is controlled by the antifungal Toll pathway, we examined gene expression in different *Drosophila* mutants after topical infection with the WT strain of *M. robertsii*. Time‐course analysis of the WT *w^1118^* flies indicated that *Sgf1* expression is highly induced upon fungal challenge (Figure 2D). However, its transcription was significantly reduced (pairwise *t*‐test: *p* < 0.01) in the *Tl^KG03609^*, *MyD88^c03881^*, *Dif^1^*, and *spz^rm7^* mutant flies (Figure 2E,F and Figure S4A). In contrast, *Sgf1* upregulation was not impaired in *Rel^E20^* flies (Figure S4B), indicating that the Imd (immune deficiency) pathway is not involved in controlling its expression upon challenge by the fungus. Notably, our attempts to generate Sgf1 antibodies were unsuccessful using either the purified protein or antigenic peptide for unclear reasons.

Next, we had the *Sgf1* gene disrupted using the CRISPR‐Cas9 technique, and two independent mutants were successfully obtained (Figure S5A). To ensure a uniform genetic background, each mutant was introgressed into the *w^1118^* line via backcrossing for at least five generations. The following survival assays revealed that *Sgf1* disruption significantly increased (*p* < 0.0001) the susceptibility of *D. melanogaster* females to either *M. robertsii* or *B. bassiana* (Figure 2H,I). Consistent with a role in host defense, higher fungal loads were observed in infected *Sgf1*‐deficient flies (Figure 2J). Taken together, our results demonstrate that the *Metarhizium* spore‐surface protein Eac1 interacts with a novel *Drosophila* factor, Sgf1, which contributes to antifungal defense.

### 2.3 Sgf1 Interacts With its Neighboring Gene to Jointly Fight Fungal Infection

We next aimed to unveil the antifungal mechanism of Sgf1. First, further Y2H screening was performed using Sgf1 as bait, and different candidate targets were obtained (Table S2). Considering that Sgf1 is an extracellular protein, only those putative secreted proteins were included in one‐by‐one verifications. As a result, Sgf1 positively targets CG30026 (Figure 3A) and CG10092 (Cathepsin B, CtsB) (Figure S5B). CG30026 is also functionally unknown and lacks any conserved domain, but exhibits slight similarity to Sgf1 (15% identity at the amino acid level; E‐value, 5e‐12). This gene was thus termed *Sgf2*. Interestingly, *Sgf1* and *Sgf2* physically cluster at the 47F10‐47F11 region of chromosome 2R of *D. melanogaster*. CtsB was also detected in interaction with the *M. robertsii* Fkp1 effector but has no contribution to antifungal response in *Drosophila* [7]. This gene was therefore not included in further analysis.

**FIGURE 3:**
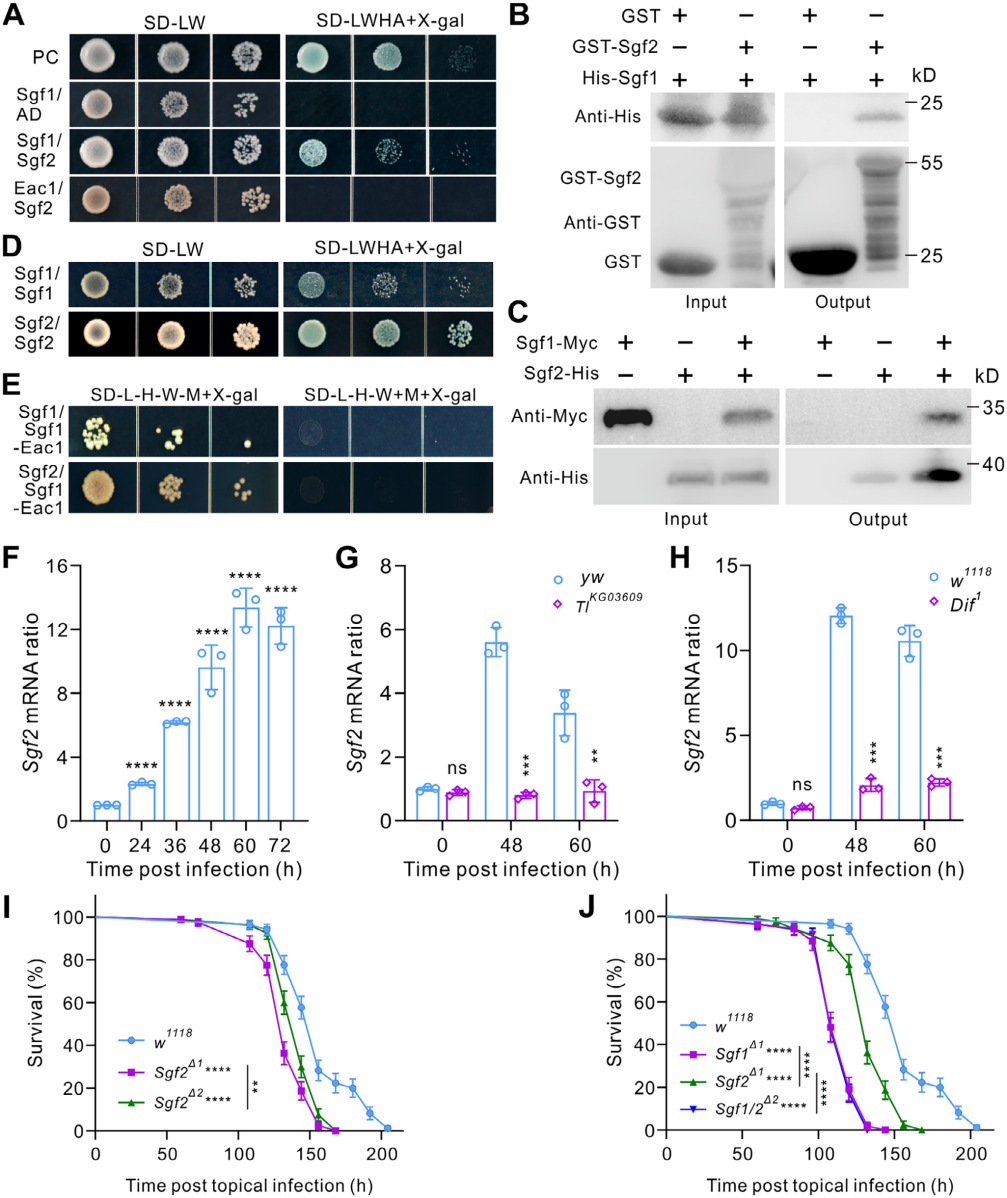
Sgf1 and Sgf2 interact with each other and with themselves to mediate antifungal defense. (A) Y2H assays show the positive interactions between Sgf1 and Sgf2 but not between Eac1 and Sgf2. (B) Protein pull‐down analysis confirms the interaction between Sgf1 and Sgf2. Original blots can be found in Figure S11C. (C) Immunoprecipitation (IP) analysis confirms the successful pull‐down of Sgf1‐Myc by Sgf2‐His after co‐expression in Sf9 cells. Original blots can be found in Figure S11D. (D) Y2H assays show the self‐interaction of Sgf1 and Sgf2. (E) Y3H assays confirm the suppression of Sgf1 self‐interaction and the interaction between Sgf1 and Sgf2 by Eac1. (F) Upregulation of *Sgf2* in *Drosophila* after topical infection with the WT *M. robertsii* for different durations. (G,H) Downregulation of *Sgf2* in the *Tl^KG03609^* (G) and *Dif^1^* (H) *Drosophila* after topical infection with the WT *M. robertsii* for different durations. Panels F‐H, data are the mean ± SD. Pairwise two‐tailed Student's *t*‐test: ^**^ *p <* 0.01; ^***^ *p <* 0.001; ns, not significant. (I,J) Independent disruptions of *Sgf2* (I) and *Sgf1/Sgf2* (J) in *D. melanogaster* significantly impair the survival of female flies following topical infection with WT *M. robertsii*. Plotted values are the mean ± SEM; Log‐rank test: ^**^ *p* < 0.01; ^****^ *p <* 0.0001.

Intriguingly, Y2H analysis showed that the fungal effector Eac1 does not interact with Sgf2 as it does with Sgf1 (Figure 3A). The His‐tagged Sgf1 and GST‐fused Sgf2 were individually expressed in *E. coli* and purified (Figure S3A). Subsequent protein pull‐down analysis confirmed the pairwise interactive relationships between Sgf1 and Sgf2 (Figure 3B). Co‐IP analysis also confirmed the positive interaction between Sgf1 and Sgf2 after being co‐expressed by baculoviruses in Sf9 cells (Figure 3C and Figure S3B). Further Y2H analysis revealed that Sgf1 and Sgf2 each self‐interact (Figure 3D), suggesting that they have the potential to form dimers or polymers. Our Y3H (yeast three‐hybrid) analysis revealed that, however, the fungal effector Eac1 suppresses Sgf1's self‐interaction and its interactions with Sgf2 (Figure 3E). RT‐qPCR analysis revealed that *Sgf2* was substantially upregulated in *Drosophila* adults after topical infection with the WT *M. robertsii* (Figure 3F). Similar to *Sgf1*, the fungus‐induced *Sgf2* expression was impaired in *Tl^KG03609^*, *Dif^1^*, *spz^rm7^*, and *MyD88^c03881^* flies but not in the *Rel^E20^* allele (Figure 3G,H and Figure S4C–E). This indicates that both *Sgf1* and *Sgf2* are regulated by the Toll pathway.

Next, for functional investigation, the *Sgf2* gene and both *Sgf1* and *Sgf2* (*Sgf1/2*) were disrupted, respectively, and two independent mutants were obtained for each allele (Figure S5C,D). Subsequent topical infection assays revealed that individual mutants of *Sgf2* and *Sgf1/2* succumbed to *Metarhizium* infection significantly faster (*p* < 0.0001) than *w^1118^* controls (Figure 3I and Figure S5E). The mutant flies *Sgf1^Δ1^*, *Sgf2^Δ1^*, and *Sgf1/2^Δ2^* (later referred to as respective mutants) were subsequently used for parallel survival assays. The results confirmed that all three mutants became significantly susceptible (*p* < 0.0001) to the WT *M. robertsii*. Furthermore, *Sgf1* is more critical than *Sgf2* (*p* < 0.0001) for antifungal defense in *Drosophila* (Figure 3J).

### 2.4 Gene Duplication and Patchy Distribution

Our BlastP analysis revealed that both *Sgf1* and *Sgf2* are drosophilid‐specific genes with a patchy distribution across *Drosophila* species. We then selected 42 *Drosophila* species, along with *Scaptodrosophila lebanonensis*, with well‐annotated genomes for phylogenomic analysis and examination of gene distribution. The obtained phylogenomic tree well separated the species into the subgenera of *Drosophila* and *Sophophora*, and the latter contains the subgroup of the Oriental lineage (Figure 4A), which is consistent with previous phylogeny analyses [26, 27]. Gene mapping revealed that *Sgf2* is present in all selected fly species except those from the *willistoni* group (*D. tropicalis* and *D. willistoni*). Within this set, only 14 species from the Oriental lineage contain both *Sgf1* and *Sgf2* (Figure 4A), suggesting that *Sgf1* might have been duplicated from *Sgf2*. In support, rebuilding the phylogeny of the Sgf1 and Sgf2 homologous proteins from these 14 species indicated that *Sgf2* diverged before *Sgf1*. Notably, *Sgf2* but not *Sgf1* is present in the basal species *S. lebanonensis* (Figure S6). We further cloned the *Sgf1‐*like gene cDNAs from *D. simulans* (DsSgf1, XP_002076296) and *D. yakuba* (DySgf1, XP_043062837), and subsequent Y2H analysis confirmed their positive interactions with Eac1 (Figure S5F). In addition, like Sgf1, both DsSgf1 and DySgf1 interact with themselves (Figure S5G). To determine their functional redundancy, we included additional *Drosophila* species that contain either both *Sgf1* and *Sgf2* (*D. simulans* and *D. yakuba*) or only the *Sgf2* gene (*D. suzukii* and *D. virilis*) for survival assays against the WT and *Eac1* mutants of *M. robertsii*. As a result, Δ*Eac1* was significantly impaired in infecting the selected species, while OE::*Eac1* was comparable to the WT strain (Figure 4B). These survival patterns were largely akin to the survival patterns of *D. melanogaster* shown above. The data suggest that Sgf1's contribution to immune defense is, to some extent, redundant in the gene‐containing flies.

**FIGURE 4:**
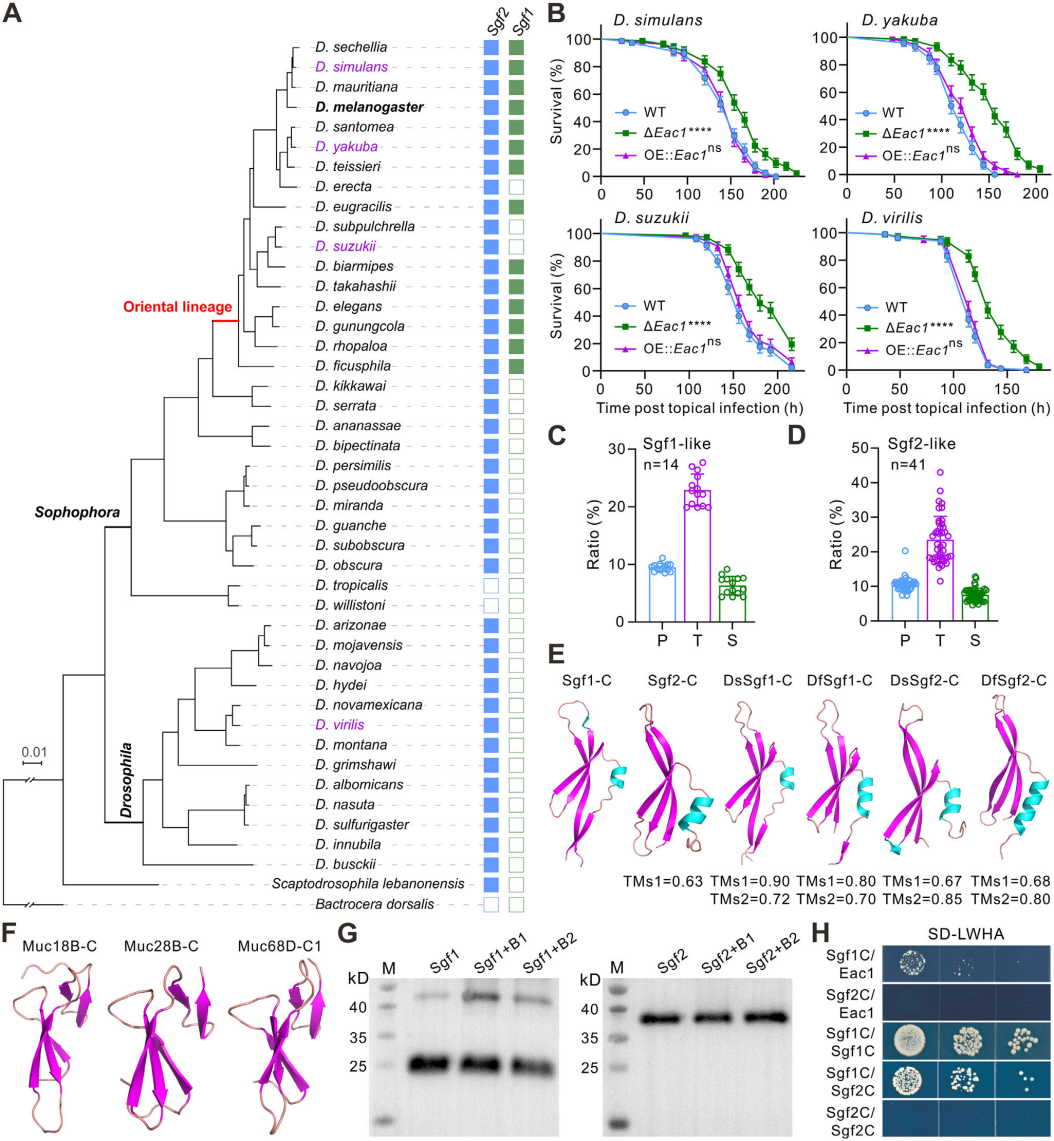
Divergent evolution and characterization of Sgf1/Sgf2 and their homologous proteins. (A) Presence/absence of Sgf1 and Sgf2 encoding genes along with *Drosophila* evolutionary speciation. Filled square, presence; open square, absence. The maximum likelihood phylogenomic tree was generated using 2,774 single‐copy orthologous protein sequences. Species labeled in purple were used for survival assays. (B) Differential survival of female flies that encode both *Sgf1* and *Sgf2‐*like genes (*D. simulans* and *D. yakuba*) or only a *Sgf2‐*like gene (*D. suzukii* and *D. virilis*) after topical infection with the WT and *Eac1* mutants of *M. robertsii*. Plotted values are the mean ± SEM. Log‐rank test between WT and individual mutant: ^****^ *p* < 0.0001; ns, not significant. (C,D) Statistics show that both Sgf1 (C) and Sgf2 (D) are rich in P (Pro), T (Thr), and S (Ser) residues. (E) C‐terminal structuring and similarity comparison among Sgf1 (9‐161 aa), Sgf2 (164‐228 aa), and their homologs in selected fly species. DsSgf1/DsSgf2, Sgf1/Sgf2 homologs from *D. simulans* (XP_002076296 and XP_016027031); DfSgf1/DfSgf2, Sgf1/Sgf2 homologs from *D. ficusphila* (XP_017038806 and XP_017038805). TMs1, TM (template modeling)‐score in reference to Sgf1; TMs2, TM‐score in reference to Sgf2. β‐sheets are labeled in magenta, and α‐helix in cyan. (F) Modeling of the type‐2 chitin binding domains (ChBDs) present in the selected *Drosophila* mucins. Muc18B‐C, the single C‐terminal ChBD of Muc18B; Muc28B‐C, the single C‐terminal ChBD of Muc28B; Muc68D‐C1, the first ChBD domain of Muc68D. (G) Deglycosylation treatment shows that the mucin‐like Sgf1 and Sgf2 are non‐glycosylated. His‐tagged proteins (6× His at C‐termini) were expressed in Sf9 insect cells, purified, and treated in buffer 1 (B1, native) and buffer 2 (B2, denaturing). Original blot can be found in Figure S11E. (H) Y2H assays show the interactive or non‐interactive relationships among the C‐termini of Sgf1/Sgf2 and fungal effector Eac1.

### 2.5 Sgf1 And Sgf2 Are Mucin‐Like Proteins

As indicated above, both Sgf1 and Sgf2 are extracellular proteins of unknown function. Our BlastP queries revealed that the Sgf1/Sgf2 homologous proteins are annotated as either integumentary mucin (e.g., XP_002033401 of *D. sechellia*) or salivary glue protein Sgs‐3‐like (e.g., XP_033155181 of *D. mauritiana*). Based on these clues, we next sought to determine whether Sgf1 and Sgf2 are mucin‐like proteins. As aforementioned, the *D. melanogaster* genome contains 30 mucin or mucin‐like proteins that are PTS‐rich with cutoff values: ST >25%, p > 0.1%, and length > 300 aa residues for each protein [23]. Owing to their smaller sizes (Sgf1, 161 aa; Sgf2, 228 aa), Sgf1 and Sgf2 were not previously identified as mucin‐like proteins. Our estimation indicated that both Sgf1 (24.8% ST and 9.3% P) and Sgf2 (34.5% ST and 9.2% P), and their orthologs, are highly PTS‐rich, with averages of 29.2% ST and 9.5% P for Sgf1 homologs (Figure 4C), and 31% ST and 10.5% P for Sgf2 homologs (Figure 4D). Similar to the reported mucins [23], the PTS‐rich region is present at the N‐terminus of each protein. Notably, the cysteine residues are all within the C‐terminal regions of these proteins, e.g., six in Sgf1 and seven in Sgf2.

We then predicted the structures of Sgf1, Sgf2, and their homologs using the AlphaFold algorithm [28] to compare their structural similarity. The N‐termini of these proteins are disordered, whereas their C‐terminal regions form three‐stranded β‐sheets, each with a confident pLDDT (predicted local distance difference test) value (90> pLDDT > 70) (Figure 4D). Pairwise comparison of these structures revealed that the obtained template modeling (TM) values were all above 0.5, indicating a high degree of structural similarity [29]. Overall, in contrast to the rather low similarity between their primary sequences as indicated above (15%), the structures of Sgf1‐C (92‐161 aa) and Sgf2‐C (164‐228 aa) are significantly similar to each other (TM = 0.63). As expected, the C‐terminal structures of Sgf1‐like proteins more closely resemble the Sgf1‐C domain than the Sgf2‐C domain of *D. melanogaster* and vice versa (Figure 4E). The results provide further support for the duplication event between *Sgf1* and *Sgf2*.

Single or multiple type‐2 chitin‐binding domains (ChBDs) are present in a few mucins or mucin‐related proteins of *Drosophila* [23]. Structure modeling revealed that these ChBDs form highly conserved three‐stranded β‐sheet structures (Figure 4F). There are certain structural similarities between these mucin ChBDs and the C‐termini of Sgf1/Sgf2, e.g., between Sgf1‐C and Muc18B‐C (TM = 0.50). Knowing that mucins are heavily glycosylated [30], we expressed both Sgf1 and Sgf2 in insect Sf9 cells, purified the proteins, and performed deglycosylation assays. The results demonstrated that the banding patterns of the two proteins were unchanged by glycosidase treatment (Figure 4G), indicating that they are not glycosylated proteins. Taken together, these data support that Sgf1 and Sgf2 are atypical mucin‐like proteins. Further Y2H analysis confirmed that, consistent with their full‐length interactions, Sgf1‐C but not Sgf2‐C positively interacts with the Eac1 effector. The interactions were also observed for Sgf1‐C self‐association, and for the Sgf1‐C/Sgf2‐C heterodimer (Figure 4H), underscoring the functional importance of the Sgf1/Sgf2 C‐terminal regions.

### 2.6 Entrapment of Fungal Spores by Sgf1 and Sgf2

Having shown above that Eac1 is a spore surface protein, we next expressed the Sgf1‐GFP‐fused protein in *E. coli*, purified it, and used it to treat conidial spores. We found that the fluorescent signal was detected on spores, indicating a bait (Eac1)‐prey (Sgf1) interaction on the spore surface (Figure 5A). Subsequently, the fungal cell components β‐(1,3)‐glucan (curdlan) and chitin, and the bacterial cell wall component peptidoglycan, were used for binding assays. As a result, Eac1 can bind chitin but not curdlan and peptidoglycan. Sgf1 and Sgf2 can bind these three polymers, and the former has stronger binding capacities (Figure 5B).

**FIGURE 5:**
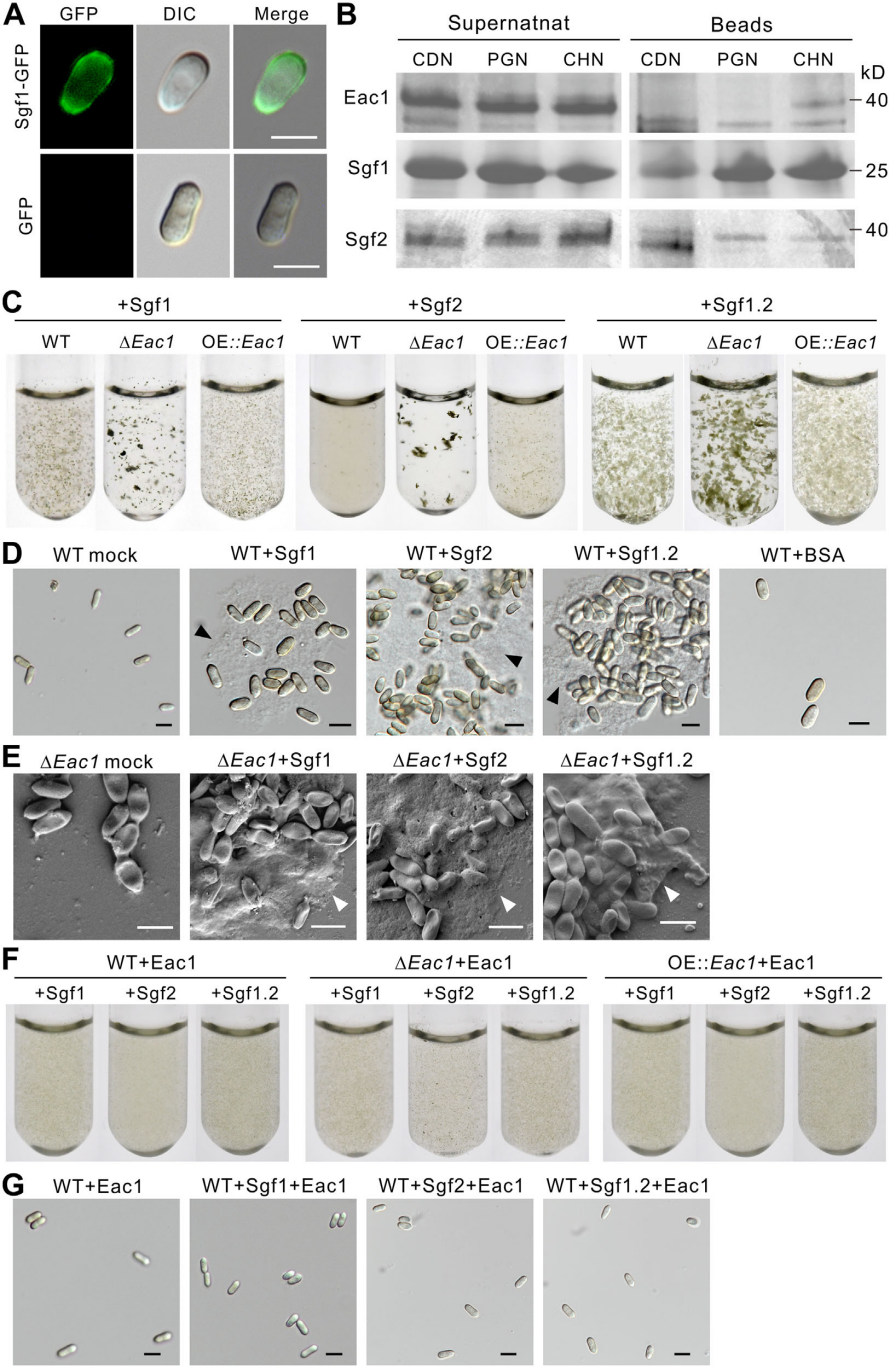
Substrate binding and fungal spore clumping mediated by Sgf1 and Sgf2, and suppressed by Eac1. (A) Sgf1 binds conidial spores. The WT conidial spores were treated with the purified Sgf1‐GFP and GFP proteins, and washed before imaging. Bar, 5 µm. (B) Differential binding of Eac1, Sgf1 and Sgf2 to different polymeric substrates. CDN, curdlan; PGN, peptidoglycan; CHN, chitin. Original images can be found in Figure S11F. (C) Differential clotting of WT and *Eac1* mutant spores by Sgf1 and Sgf2 in glass tube assay. (D) Light microscopic imaging shows the entrapment of WT spores by Sgf1 and Sgf2. BSA (bovine serum albumin) was used at the same amount as a negative control. (E) Scanning electron microscopic images show the clumping of the Δ*Eac1* spores by Sgf1 and Sgf2. The formation of colloid‐like protein matrix is arrowed in panels D and E. (F,G) Eac1 addition substantially reduces the Sgf1/Sgf2‐mediated clumping of WT and *Eac1* mutant spores as shown by glass tube assay (F) and microscopy (G). Bar, 5 µm. Spore suspensions (5 × 10^7^ conidia/mL in 0.05% Tween 20) were individually incubated with the purified protein (0.5 mg/mL) at 4°C for overnight, gentle shaking and imaging.

Next, we prepared conidial spore suspensions of the WT and *Eac1* mutants in 0.05% Tween 20 (Figure S7A), and the addition of Sgf1 and Sgf2 individually or in combination led to the formation of spore clumps, especially for the Δ*Eac1* spores (Figure 5C). Light microscopic observations indicated that, in contrast to mock controls (without Sgf1/Sgf2 or with bovine serum albumin), the WT conidia were glued and entrapped in colloidal‐like matrices formed by Sgf1/Sgf2, i.e., the mucin gel‐like assembly (Figure 5D). Further scanning electron microscopy analysis using the Δ*Eac1* conidia confirmed the formation of gel matrices by Sgf1, Sgf2, or the mixture of Sgf1 and Sgf2 (Figure 5E). Cysteine residues are essential for the structure and function of gel‐forming mucins [31]. In consistency, our tests using dithiothreitol and β‐mercaptoethanol highly reduced the clotting activity of Sgf1 and Sgf2 against Δ*Eac1* conidia (Figure S7B,C). The results further supported that both Sgf1 and Sgf2 are mucin‐like proteins. In support of the above Y3H analysis, the addition of Eac1 blocked the Sgf1‐ and Sgf2‐mediated formation of the WT conidial clumps (Figure 5F,G). Clumping assays using yeast‐like hyphal‐body cells harvested from the wax moth body cavity revealed that neither Sgf1 nor Sgf2 induced clumping of cells from either the WT or Δ*Eac1* strain (Figure S7D). Furthermore, we observed that *B. bassiana* conidia could be clotted by Sgf1 and Sgf2, whereas the conidia of the non‐entomopathogenic fungus *Aspergillus fumigatus* were not entrapped by either Sgf1 or Sgf2 (Figure S7E). Collectively, Sgf1 and Sgf2 form colloidal‐like gels to trap pathogenic fungal conidia; however, this entrapment can be blocked by the fungal effector Eac1.

Even showing peptidoglycan‐binding activities, both Sgf1 and Sgf2 did not aggregate the cells of either the Gram‐positive (e.g., *Enterococcus faecalis*) or the Gram‐negative (e.g., *E. coli*) bacteria (Figure S8A). Survival challenge with the Gram‐negative *Erwinia carotovora carotovora* 15 (*Ecc15*) caused a survival difference in *Sgf2^Δ1^* and *Sgf1/2^Δ2^* females but not in *Sgf1^Δ1^* females (Figure S8B). No difference in survival was observed between the *w^1118^* and *Sgf1/2^Δ2^* alleles following injection with *A. fumigatus* spores (Figure S8C).

### 2.7 Differential Immune Responses of Mutant Flies to Fungal Challenge

To examine cellular immune response within the insect body cavity, we next injected the wax moth larvae with the WT and *Eac1* mutants of *M. robertsii*. In line with fungal comparable infectivity shown above, multiple WT and mutant spores were similarly entrapped to form nodules by hemocytes. Subsequently, the hyphae escaped and formed large numbers of hyphal‐body cells within 36 h (Figure S9). However, our injection of female flies with the GFP‐labeled *M. robertsii* conidia demonstrated that multiple spores/germlings were tightly entrapped by the *w^1118^* hemocytes. By contrast, large numbers of hyphal‐body cells formed in *Sgf1^Δ1^*, *Sgf2^Δ1,^* and *Sgf1/2^Δ2^* body cavities within 36 h post‐challenge (Figure 6A). The following intravital microscopy imaging of anesthetized flies showed that, unlike the sparse distribution of fluorescent fungal cells in *w^1118^* flies, large numbers of fungal cells were observed in *Sgf1* and *Sgf2* mutant flies. This was especially pronounced in the *Sgf1/2^Δ2^* double mutant at the same time point post‐challenge (Figure 6B). Fungal load assays confirmed the presence of significantly higher levels of fungal cells (One‐way ANOVA, *p* < 0.01) in mutant flies, particularly in the double mutant, compared to those in *w^1118^* (Figure 6C,D). Our measurement of hemolymph phenoloxidase (PO) activity revealed that *Sgf1^Δ1^* had the highest PO activity, followed by *Sgf1/2^Δ2^*, whereas *w^1118^* and *Sgf2^Δ1^* had similar levels of PO activity following topical infection with the WT *M. robertsii* for 48 h (Figure 6E)

**FIGURE 6:**
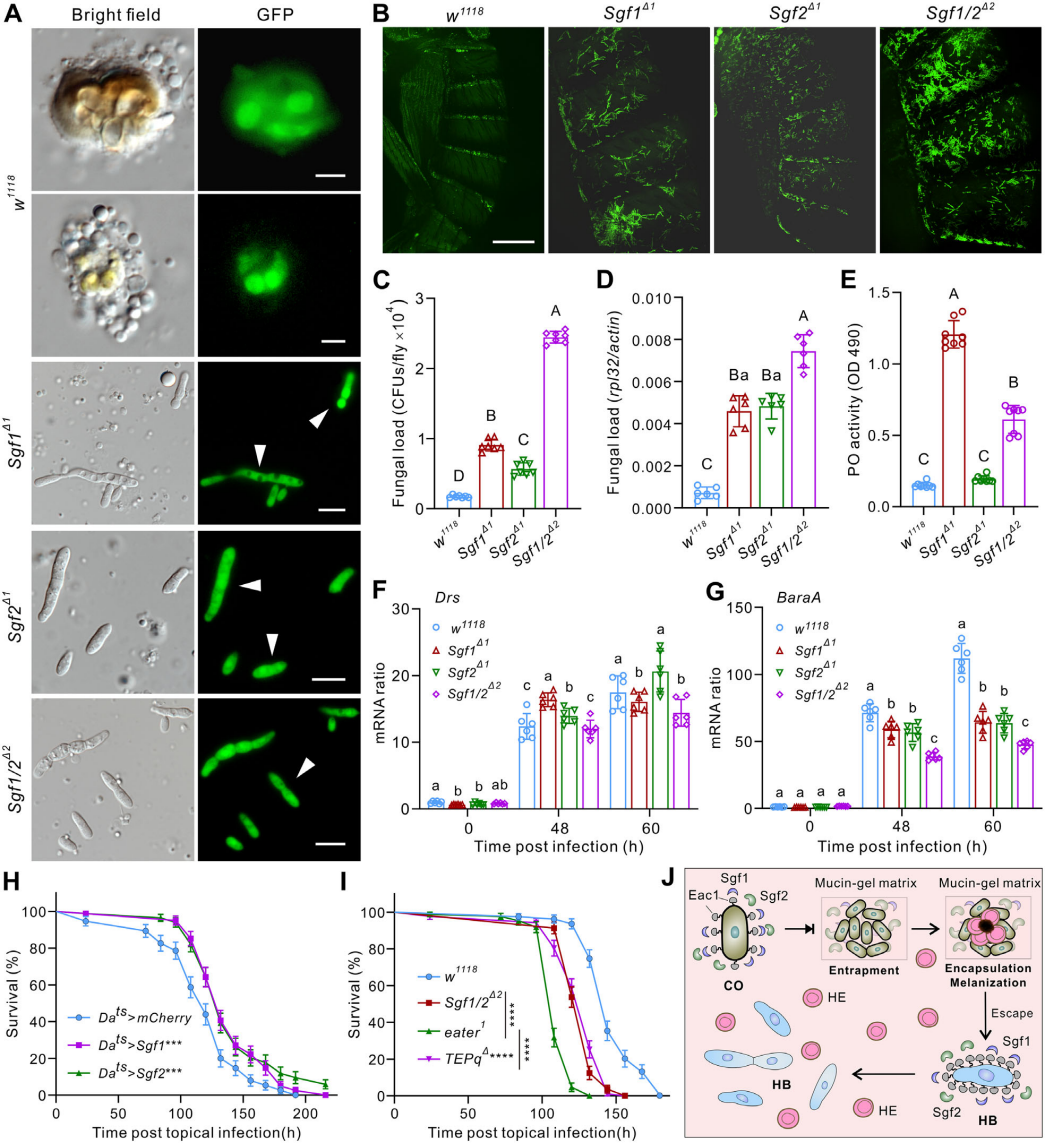
Differential antifungal immune defense among *Drosophila* female lines following fungal challenge. (A) Microscopic examination of fungal development in the WT and mutant flies. Flies were injected with the GFP‐labeled spores of *M. robertsii* for 36 h. Unlike those fungal cells entrapped in *w^1118^*, hyphal‐body cells (arrowed) were formed within the body cavity of mutant flies the same time post‐treatment. Bar, 5 µm. (B) Intravital imaging shows fungal cell propagation within the fly body cavities. Flies were injected with the GFP‐labeled conidia of *M. robertsii* for 48 h. Bar, 80 µm. There were at least five independent replicates, and representative images are presented. (C,D) Fungal load assays of different female mutants by cell counting (C) and qPCR analysis (D) 36 h post‐injection with the WT spores. CFU, colony‐forming unit. (E) Variation of hemolymph PO activity between *w^1118^* and mutant flies. Hemolymph samples were collected from female flies 48 h post‐topical infection with the WT *M. robertsii*. (F,G) Differential expression of the antifungal genes *Drs* (F) and *BaraA* (G) in female mutants after topical infection with the WT *M. robertsii* for different durations. Panels C‐G: data are the mean ± SD; One‐way ANOVA followed by the Tukey's tests among samples: different capital letters, *p <* 0.01; different lower letters, *p <* 0.05. (H) Overexpression of *Sgf1* or *Sgf2* significantly reduces female fly susceptibility to topical infection with WT *M. robertsii*. (I) Differential survival of *Sgf1/2^Δ^* ^2^, *eater^1^*, and *TEPq* ^Δ^ female flies after topical challenge with WT *M. robertsii*. Panels H and I: plotted values are the mean ± SEM. Log‐rank test: ^***^ *p* < 0.001; ^****^ *p* < 0.0001. (J) Schematic of the interaction between the fungal effector Eac1 and fly mucin‐like proteins Sgf1/Sgf2 in determining fungal cell entrapment and anti‐entrapment. Fungal spores are entrapped in the Sgf1/Sgf2 mucin‐gel matrix first before being encapsulated and melanized by hemocyte (HE) cells. However, the Eac1‐coated hyphal‐body (HB) cells escape attacks by Sgf1 and Sgf2 and freely live in the host body cavity. CO, conidium. The arrow‐to‐bar symbols mean the combined effect of induction and inhibition mediated by Eac1.

Having shown the evident defects of cellular immunity after disruption of *Sgf1* and *Sgf2* in *Drosophila*, we examined the expression of defense peptide genes following topical infection with the WT *M. robertsii*. As a result, reduction of *Drs* expression was only observed (*p* < 0.05) in *Sgf1/2^Δ2^*, whereas *BaraA* expression was significantly reduced in three mutant flies compared to *w^1118^* (Figure 6F,G). The expression of *Mtk* was also only downregulated in *Sgf1/2^Δ2^*, and the *Dso1* and *BomS1* expressions were largely comparable between the *w^1118^* and mutant flies (Figure S10A–C). This indicates that *Sgf1* and *Sgf2* are not required for AMP gene expression.

We also had *Sgf1* and *Sgf2* individually overexpressed in *D. melanogaster*, and the transgenic flies survived better (*p* < 0.001) to *Metarhizium* infection (Figure 6H). Finally, to compare with those known cellular immune factors such as Eater and TEPs [15, 18], we performed parallel survival assays using the WT strain of *M. robertsii* to infect the *Sgf1/2^Δ2^*, *eater^1^* and *TEPq* ^Δ^ (lacking *TEP1‐4*) mutant lines. All mutant flies succumbed to fungal infection much faster than *w^1118^* (*p <* 0.0001). The survival curves of *Sgf1/2^Δ2^* and *TEPq* ^Δ^ mutants were similar, whereas *eater^1^* died significantly faster (*p* < 0.0001) (Figure 6I), suggesting that Sgf1/2 play a role in cellular immunity equivalent to that of TEP1‐4. Interestingly, both *Sgf1* and *Sgf2* were upregulated in *eater^1^* and *TEPq* ^Δ^ flies following *Metarhizium* challenge (Figure S10D–G), implying that *Sgf1* and *Sgf2* expression is not under the control of *eater* or *TEP* genes but instead mediates a compensatory effect in the *Drosophila* immune response.

## 3 Discussion

In this work, we identified a spore surface protein, Eac1, required for full virulence in *M. robertsii*. Eac1 binds Sgf1 but not Sgf2. Both Sgf1 and Sgf2 are atypical mucins encoded by the clustered genes in *D. melanogaster*. These proteins are specific to drosophilids, with *Sgf1* likely originating from a duplication of *Sgf2*. These two proteins each self‐interact and interact with each other, exhibiting cell‐wall component binding and gel‐forming activities that trap fungal spores; however, their functions can be effectively blocked by the cell surface Eac1. Intriguingly, Sgf1 and Sgf2 do not clot the conidia of the non‐pathogenic fungus *A. fumigatus*, bacterial cells, and the hyphal‐body cells of *M. robertsii* formed within the insect body cavity. Gene disruptions in *Drosophila* and subsequent infection assays revealed that individual mutants of *Sgf1* and *Sgf2*, and especially the double mutant, were significantly impaired in defenses against fungal infection. Our data reveal that the entrapment of multiple fungal spores by *Drosophila* atypical mucins is a critical step for the hemocyte‐mediated encapsulation/nodulation and melanization (Figure 6J).

Spore surface proteins play essential roles in the interface of fungal infection and immune evasion [32, 33]. The collagen‐like coat protein Mcl1 of *M. anisopliae* has been demonstrated to function in evading the encapsulation of caterpillar hemocytes by masking cell wall carbohydrates [34]. The conidial surface protein Mcdc9 of *M. robertsii* can be detected by a *Drosophila* chemosensory factor, which triggers behavioral immunity in flies [25]. In this work, the GPI‐anchored Eac1 was identified on the surfaces of conidial and hyphal‐body cells, where it blocks nodulation mediated by host atypical mucins. Interestingly, *Eac1* is only present in *Metarhizium* species and required for virulence specifically during the infection of *Drosophila* but not during infection of wax moth and silkworm larvae. The absence of a homologous target gene in caterpillars may help explain why there were no overt differences in killing caterpillars between the WT and *Eac1* mutants of *M. robertsii*. Likewise, the drosophilid‐specific immune factor Dsff1 is targeted by the *B. bassiana* strain‐specific effector Bhe1, and the presence or absence of *Bhe1* dictates the variation in virulence among *B. bassiana* isolates against flies [9]. Eac1 was not detected on the newly‐formed appressoria and mycelia growing in an artificial liquid medium. Differential regulation of *Eac1* in *M. robertsii* remains to be determined.

Based on a previous estimation of gene age in *Drosophila*, *Sgf1* is a new gene belonging to the phylogeny branch‐4 [originated ca. 33 million years ago (MYA)], whereas *Sgf2* is the branch‐0 old gene (emerged ca. 69 MYA) [35]. These dating data support our assumption that *Sgf1* was duplicated from *Sgf2*. *Sgf1* is missing in a few species such as *D. suzukii*, and its emergence only occurred in the Oriental lineage of *Drosophila*. Including the diversified *D. melanogaster* lines, the Oriental lineage represents one of the major global centers of drosophilid diversity and has broader microbial associations [36, 37]. We found that *Sgf1* plays a more essential role than *Sgf2* in defense against fungal infection. This suggests that fly species of the Oriental lineage might have faced stronger selective pressure from fungal parasites, driving the gene duplication and functional divergence of *Sgf1*. Likewise, the duplicated genes in *Drosophila*, such as the odorant‐binding proteins *Obp50* paralogs and the antibacterial Diptericin genes *DptA/DptB*, have undergone functional diversification and even neofunctionalization [38, 39]. The absence of *Sgf1* and *Sgf2* in the *willistoni* group of *Drosophila* is still enigmatic. The lack of *Sgf1* in three Oriental species (e.g., *D. suzukii*) suggests either that gene duplication did not occur or that a gene gain event was followed by loss.

Neither Sgf1 nor Sgf2 was classified as a mucin or mucin‐like protein because their lengths fall below the required cutoff of >300 aa [23]. Our experiments demonstrated that, although non‐glycosylated, both proteins are PTS‐rich, contain multiple cysteine residues, and exhibit gel‐forming features that are disruptable by reducing agents. Taken together, both proteins are mucin‐like but atypical. Both proteins bind fungal cell wall components (β‐glucans and chitins) and entrap the conidial spores of both *Metarhizium* and *Beauveria* species, which is supported by containing a putative carbohydrate‐binding‐like domain in each C‐terminus. Although the Δ*Eac1* spores were more severely clotted by Sgf1/Sgf2, both the WT and even OE*::Eac1* spores could still be entrapped by both proteins. These findings suggest that the amount of Eac1 coated on conidial spores might be insufficient to fully counteract the clotting activity of Sgf1 and Sgf2. On the other hand, intriguingly, neither Sgf1 nor Sgf2 clots the hyphal‐body cells of both the WT and Δ*Eac1* of *M. robertsii*. Extensive remodeling of the fungal cell wall occurs upon penetration of the insect cuticle and entry into the hemocoel, including a sharp reduction in surface β‐glucans that likely helps evade host immunity [40, 41, 42]. Taken together with the coating of Eac1 on the hyphal‐body cell surface, Sgf1 or Sgf2 becomes incapable of binding these cells and entrapping them. It is enigmatic why both proteins fail to clot *Aspergillus* conidia yet effectively entrap *Beauveria* spores, given that both spore types lack an Eac1‐like protein. This specificity suggests that these two atypical mucins might function, at least in part, as pathogen‐specific effectors capable of discriminating between spore types. Likewise, the receptor Eater displays varied binding affinities for distinct classes of bacteria [16]. It also remains unclear why both proteins do not entrap both Gram‐positive and Gram‐negative bacterial cells, even though they bind peptidoglycan.

As shown in this study and reported elsewhere [34, 43], multiple fungal spores are frequently clotted together within individual hemocyte nodules. It is a rare case of nodule formation found in *Drosophila* adults, in which hemocytes are mostly sessile [44]. Our biochemical assays revealed that pathogen spores were entrapped in the Sgf1/Sgf2‐gel matrix, a mechanism that has not been suspected before. Multiple fungal spores were also trapped in the hemocyte nodules of the *Sgf1*/*Sgf2‐*absent wax moth larvae, suggesting that caterpillars have a distinct strategy to entrap fungal spores for encapsulation. Indeed, Noduler, a reeler‐domain‐containing extracellular matrix protein identified in the silkmoth *Antheraea mylitta*, binds to bacterial and fungal cell wall components to mediate nodulation [45]. Our in vivo assays revealed that null mutants of *Sgf1*, *Sgf2*, and *Sgf1/2* were impaired in encapsulation of fungal spores and defense against fungal colonization. However, the null mutants of *Sgf1* and *Sgf2* differed significantly in survival and PO activity following fungal challenge, suggesting that the functions of these two genes are not fully equivalent or functionally redundant. Different modules of immune responses are interconnected or compensated [8, 11]. Higher levels of PO activity in *Sgf1^Δ1^* and *Sgf1/2^Δ2^* flies suggest that more spores became unentrapped before encapsulation in these mutants, thereby triggering a stronger melanization response. Elevated melanin production is toxic to both the fungal pathogen and the insect host [46]. This difference likely explains, at least in part, why *Sgf1^Δ1^* flies succumbed to fungal infection faster than *Sgf2^Δ1^* flies. Nevertheless, functional divergence between Sgf1 and Sgf2, including the magnitude of their difference in spore clotting activity, requires further investigation.

Together with the recent works [6, 7, 8, 9], this study further establishes the occurrence of gene‐for‐gene interaction between fungal entomopathogens and their insect hosts. Eac1 targets Sgf1 while the latter binds Sgf2. Both Sgf1 and Sgf2 have spore entrapment activities; however, their interaction and clotting function are subverted by Eac1, reflecting a pattern reminiscent of the arms‐race evolution between pathogens and hosts [12]. The absence of *Eac1*, as well as the hijacking effector *ETS6* [8], in the early‐diverged *Beauveria* species, would suggest that *Metarhizium* fungi have evolved novel virulence factors/effectors later to more effectively cope with diverse insect hosts. Notably, as with its defective infection of *Sgf1*/*Sgf2‐*present flies, the *M. robertsii* Δ*Eac1* mutant was also impaired in infecting the *Sgf2‐*only species *D. suzukii* and *D. virilis*. Rather than directly interacting with Sgf2, Eac1 may suppress its activity by functioning as a masking protein coat on spore surfaces, which blocks Sgf2‐mediated binding and entrapment. The intricate relationships among the effector Eac1, its target Sgf1, and the non‐target Sgf2 require further investigation.

Our parallel survival data revealed that, unexpectedly, *eater^1^* flies were mostly susceptible to *Metarhizium* infection, while the survivals of *Sgf1/2^Δ2^* and *TEPq^Δ^* were comparable. The data therefore confirm that Eater, being essential for phagocytosis of bacteria [15], is also required for defense against fungi as reported [47]. In contrast, a recent work showed that the double mutant *NimC1^1^;Eater^1^* had a comparable survival pattern to that of *w^1118^* following natural infection with *B. bassiana* or *Metarhizium* species [48]. We found that both *Sgf1* and *Sgf2* are regulated, at least primarily, by the Toll pathway. Notably, even though it was not obviously controlled by the Imd pathway, CG34054 (Sgf1) was detected as an early‐induced protein in the *Drosophila* hemolymph after injection with the Gram‐positive bacterium *Micrococcus luteus* or with the Gram‐negative *Ecc15* [49]. Interestingly, the upregulations of *Sgf1*/*Sgf2* were detected in *eater^1^* and *TEPq* ^Δ^ flies. These three groups of cellular immune factors may play compensatory roles in protecting flies against fungal infection.

In conclusion, we have identified a fungal spore‐coat protein that subverts entrapment by *Drosophila* atypical mucins. Our findings advance the understanding of insect cellular immunity and the evolutionary arms race between entomopathogens and their insect hosts.

## 4 Experimental Section

### 4.1 Microbial Cultures and Maintenance

The WT fungal strain ARSEF 23 of *M. robertsii* [50] was used for genetic transformation. In addition, the strains of *B. bassiana* ARSEF 2860 and *A. fumigatus* Af293 (Table S3) were used for transgenesis, insect infection, or spore clumping assays. Fungal cultures were maintained on PDA (BD Difco) at 25°C for two weeks for spore harvesting. Fungal cultures were also grown in the liquid Sabouraud dextrose broth (SDB, BD Difco) for RNA extraction and gene expression analysis after incubation in flasks at 200 rpm and 25°C. The spores of *M. robertsii* were either induced on a hydrophobic surface or on black soldier fly (*Hermetia illucens*) wings for 18 h [7]. The last‐instar wax moth larvae were injected with the WT *M. robertsii* spore suspension (10 µL each of 2 × 10^7^ conidia/mL), and the hyphal‐body cells were harvested for RNA extraction 36 h post‐injection by gradient centrifugation in Percoll (Sigma–Aldrich) at 120 00 rpm and 4°C for 15 min. The pellet was washed twice with 1 mL phosphate buffer saline (pH = 7.0) before RNA extraction using a TRIzol reagent (Thermo Fisher Scientific). The budding yeast *Saccharomyces cerevisiae* strains Y2H Gold and AH109 were used for Y2H and Y3H analysis, respectively. Yeast strains were grown on the synthetic dropout (SD) media (Sigma‐Aldrich). Different strains of *E. coli* were grown on Luria‐Bertani (LB) agar or in LB broth used for gene cloning (Top10, Weidi Biotech) at 37°C or protein expression (Rosetta) at 16°C. The AGL1 strain of *Agrobacterium tumefaciens* was maintained at 28°C and used for fungal transformation. The Gram‐negative *Ecc15* was maintained on LB agar and used for the injection of flies for survival assays.

### 4.2 Fly Stocks and Insect Rearing

Different mutants of *D. melanogaster* were used in this work, including the WT *w^1118^* (BDSC: 5905) and A5001, and mutant lines *spz^rm7^*, *Tl^KG03609^* (BDSC: 13022), *MyD88^c03881^* [51], *Dif^1^* and *Rel^E20^*, as well as the *Sgf1* and *Sgf2* mutant lines generated in this study (see below). Tool lines *w*;*Da‐Gal4;tub‐Gal80^ts^* and *w;Ubi‐Gal4;tub‐Gal80^ts^* were used to drive the expression of transgenic genes in *Drosophila* (Table S3). Cellular immunity‐related stocks *eater^1^* and *TEPq* ^Δ^ were also included in comparative survival assays. Flies were routinely maintained at 25°C and 12 h of light/dark cycles on the Bloomington formulation of cornmeal agar [52]. In addition, other fly species, including *D. simulans* (BCF #93)*, D. yakuba* (BCF #94), and *D. virilis* (BCF #97) from the Core Facility of Drosophila Resource and Technology, Chinese Academy of Sciences; the spotted‐wing drosophila *D. suzukii* [7], the wax moth (*G. mellonella*), and silkworm (*Bombyx mori*) larvae were used for survival assays. *Drosophila* stocks were maintained at 25°C and 12 h of light/dark cycles on the Bloomington formulation of cornmeal agar medium. Silkworm eggs were hatched on filter paper, and larvae were reared in a growth chamber at 25°C with fresh mulberry leaves, provided every 12 h [53]. The last‐instar wax moth larvae were ordered from a fishing bait company (Ruiqing, Shanghai) and maintained in Petri dishes on filter paper (as food) at 25°C and 50% relative humidity.

### 4.3 Eac1 Localization Assays

Eac1 was predicted as a putative effector‐like protein using EffectorP 3.0 [54], and the C‐terminus of the secretable Eac1 contains a GPI anchor site [55]. To verify its localization feature, we generated the construct Pro_Eac1_::*SP‐GFP‐Eac1* in the binary plasmid pDHt‐Bar, i.e., the in‐frame fusion of the GFP gene between the signal peptide and mature protein under the control of its self‐promoter. The vector was used for the *Agrobacterium‐*mediated transformation of the WT *M. robertsii* strain. The previously obtained GFP‐labeled strain was used as a control [56]. The expression and localization of Eac1 were examined on conidial spores, appressoria, hyphal bodies, and mycelia harvested from the artificial medium SDB. The fluorescent dye Calcofluor white (Sigma–Aldrich) was used to stain cell wall chitins for the merging of protein localization. The dye was prepared in distilled water to a final concentration of 0.1% (w/v). Fungal samples were placed on a glass slide, and one drop of solution was added. After placing a coverslip, each sample was incubated for 5 min at room temperature in the dark. Samples were examined under a fluorescent microscope (Nikon, DS‐Ri2).

### 4.4 Fungal Gene Deletion, Overexpression, and Mutant Characterizations

Deletion of *Eac1* in *M. robertsii* was conducted by homologous replacement and *Agrobacterium*‐mediated transformation as previously described [57]. In brief, the 5ʹ‐ and 3ʹ‐flanking regions (1–1.5 kb) of *Eac1* were amplified by PCR using different primer pairs (Table S4). The products were purified and subsequently cloned into the binary vector pDHt‐bar (conferring resistance to ammonium glufosinate) for transformation of the WT strain of *M. robertsii*. For overexpression of *Eac1*, the open reading frame of this gene was amplified and cloned into the binary plasmid pDHt‐Sur (conferring sulfonylurea resistance) under the control of the constitutive *Tef* promoter for *M. robertsii* transformation to obtain OE::*Eac1* strains [58] or the *GpdA* promoter for *B. bassiana* transformation to obtain Bb::*Eac1* strains [59]. The drug‐resistant mutants were selected for single spore isolations and verified by PCR and/or RT‐PCR with different primers (Table S4).

The WT and verified mutants of Δ*Eac1* and OE::*Eac1* were examined for appressorium induction on the hydrophobic surface of Petri dishes (6 cm in diameter) containing the minimum medium [60]. Spore suspensions of each strain were individually inoculated (2 µL of 5 × 10^6^ conidia/mL) in the middle of cicada (*Cryptotympana atrata*) wings that were placed on 0.7% agar plates. The adult cicadas were collected from the field in summer, and the wings were dissected and stored in a freezer (−20°C) before use. After incubation for two days (upper panels), the wings were removed, and the plates were incubated for five additional days (lower panels) [61]. Having shown that Eac1 is a spore surface protein, we examined the effect of *Eac1* deletion and overexpression on conidial spore hydrophobicity using hexadecane as described previously [62].

### 4.5 Insect Survival Assays

The obtained *Eac1* mutants were used with the WT strains of *M. robertsii* and *B. bassiana* for infection assays against different insects. Thus, the spore suspensions (5 × 10^5^ conidia/mL in 0.05% Tween 20) of the WT and *Eac1* and OE::*Eac1* of *M. robertsii* were prepared and used for the topical infection of *D. melanogaster* and *D. suzukii* females (3 days post eclosion; 80 insects per sample). The increased concentration of spore suspension (1 × 10^7^ conidia/mL) was prepared to topically infect the last‐instar larvae of wax moth (50 insects per treatment). For topical infections, female flies were anesthetized with CO_2_, while the wax moth and silkworm larvae were anesthetized on ice for 30 min. The anesthetized insects were immersed in spore suspensions for 30 sec, with 0.05% Tween 20‐treated insects serving as mock controls. The treated insects were kept in a high‐moisture condition (relative humidity > 95%) for 24 h and then maintained in a growth chamber at 25°C [25]. In addition, spore suspensions were prepared to inject female flies (10 nL each of 5 × 10^6^ conidia/mL) using a nanoinjector (Nanoject III, Drummond, Broomall, PA). The fifth‐instar larvae of silkworms (60 per experiment) were also injected (5 µL each of 5 × 10^4^ conidia/mL) using a microapplicator (Burkard, Hertfordshire, UK). The WT and Bb::*Eac1* spore suspensions (2× 10^7^ conidia/mL) were also prepared for the topical infection of *D. melanogaster* females.

The obtained mutants (described below) of *D. melanogaster* were examined for survival after topical challenge with the WT strain of *M. robertsii*. In addition, the spore suspension of *A. fumigatus* was used to inject the WT and mutant flies (10 nL each of 5 × 10^8^ conidia/mL). In this case, the WT A5001 and *MyD88^c0388^* mutant flies were included as controls. Insect mortality was recorded every 12 h for each sample. The Gram‐negative *Ecc15* was also used for injection of the WT and mutant flies (25 nL each of OD600 = 6). Each experiment was repeated at least twice.

### 4.6 Targeted Protein Screening and Verification

To determine the *Drosophila* target of Eac1, we performed a screening of a yeast library containing the cDNAs of *D. melanogaster* after fungal immune challenge [25]. Thus, *Eac1* cDNA was cloned without its signal peptide region into the bait plasmid pGBKT7. The generated vector was transformed into the yeast strain Y2H Gold by following the standard protocols [25]. The transformed cells were inoculated on the SD‐Trp agar (TaKaRa; Table S3), and the positive clones were collected, propagated, and further transformed with the pGADT7 library plasmids. Positive clones were selected on the SD‐Trp‐Leu‐His‐Ala (TaKaRa Bio) agar with or without X‐Gal (Yeasen) [63]. The plasmids were then extracted from the yeast cells for sequencing. The full cDNA of the putative targets was then cloned into the pGADT7 vector for Y2H verification by following the manufacturer's protocols (Takara Bio, 630489). Y3H analysis was performed using the pBridge vector (Clontech, TaKaRa Bio) to determine whether Eac1 contributes to blocking the interaction between Eac1 and Sgf2, as we did before [7].

### 4.7 Protein Expression and Interaction Analysis

To further confirm the interactions between proteins, we performed the heterologous expression of the C‐terminal GST‐, His (6×)‐ or GFP‐tagged proteins in *E. coli*. cDNA of *Eac1, Sgf1*, *Sgf1‐GFP* fused cassette, and *Sgf2* were individually cloned in fusion with an alternative tag in the plasmid pET‐28b (for His‐tagged protein) or pGex‐6p‐1 (Addgene; GST‐tagged protein) for transformation of the competent Rosetta cells of *E. coli*. A single drug‐resistant colony was transferred into a test tube containing 4 mL of LB medium. After incubation for 6–8 h at 160 rpm at 37°C, 1 mL of each culture was transferred into the 250 mL flask containing 100 mL of LB medium with the addition of 20 µL of 1 M isopropyl β‐D‐1‐thiogalactopyranoside (IPTG; Beyotime). The GST protein was also expressed and purified for use as a control. After incubation overnight at 18°C and 120 rpm, cells were harvested by centrifugation and disrupted with the One‐Shot cell Disruptor (Constant Systems, UK) for protein purification using Glutathione Sepharose (GS, TransGen Biotech) and Ni‐NAT Superflow Agarose (SMART Life Sciences) for GST‐ and His‐tagged proteins, respectively. Protein pull‐down analyses were conducted in the binding buffer (50 mM Tris‐HCl, pH 8.0; 150 mM NaCl; 1 mM phenylmethylsulfonyl fluoride) for incubation at 4°C with gentle shaking for 3–4 h [25]. In addition, the fused Sgf1‐Myc (a peptide tag, EQKLISEEDL, derived from the human c‐Myc protein) and Eac1‐His, and Sgf1‐Myc and Sgf2‐His were co‐expressed in Sf9 cells (detailed below) for Co‐IP analysis to confirm protein interactions, respectively. The fused GFP‐Sgf1‐His protein was expressed, purified, and used for binding of the *M. robertsii* conidial spores. Insoluble (inclusion‐body) proteins were obtained for those His‐tagged expressions, which were reconstituted based on the standard protocol [64].

### 4.8 Gene Disruption in D. Melanogaster and Phenoloxidase Activity Assay

The *Sgf1*, *Sgf2*, and *Sgf1/Sgf2* double gene‐disruption mutant flies of *D. melanogaster* were generated at the National Drosophila Resource Center of China (http://ndrcc.sibcb.ac.cn/ndrcc/) using the CRISPR‐Cas9 technique [7]. Two sgRNAs each were designed and used for the disruption of *Sgf1* (Sgf1Sg1 and Sgf1Sg2) and *Sgf2* (Sgf2Sg1 and Sgf2Sg2), respectively (Table S4). Since *Sgf1* and *Sgf2* are clustered together, double deletion of these two genes was generated using the embryos of the isogenic *Sgf1^Δ1^* for further deletion of *Sgf2* using three sgRNAs (DKOSg1, Sgf2Sg1, and Sgf2Sg2) (Table S4). Each mutant was backcrossed into a *w^1118^* background for more than five generations, followed by two to three generations of sibling crosses to remove balancers before phenotypic analysis [6]. Gene disruption was verified by PCR and PCR band sequencing, and two independent mutant lines were used for each gene and double gene disruptions.

To determine whether there is any association between the Sgf1/Sgf2 functions and melanization, we performed a PO activity assay. Thus, female flies (approximately 700 each) of *w^1118^* and mutants were topically infected (5 × 10^5^ conidia/mL) for 48 h. Hemolymph samples were collected by centrifugation [6]. Protein concentration was adjusted to 15 µg/µL, and 150 µL of each sample was mixed with 450 µl L‐DOPA (5 mM; SparkJade), which was mixed and incubated at 29°C for 30 min. Aliquots of 100 µl sample were subsequently transferred into the 96‐well plate for recording their absorbance values at a wavelength of 490 nm [65].

### 4.9 Phylogenetic Analysis

To determine the distribution of *Sgf1* and *Sgf2* in *Drosophila*, we selected 42 representative *Drosophila* species plus *Scaptodrosophila lebanoenesis* with well‐annotated genome information [27, 66]. The tephritid fruiting fly *Bactrocera dorsalis* was selected as an outgroup. For phylogenomic analysis, 2774 single‐copy orthologous protein sequences were identified from each species using OrthoFinder (version 3.0.1b1) [67]. Orthologous protein sequences were aligned individually with MAFFT (version 7.525), and the alignments were trimmed with the program trimAl (version 1.4.rev15). The trimmed alignments were concatenated into a single sequence alignment (comprising 44 taxa) using a custom Perl script. A maximum‐likelihood phylogenetic tree was inferred using IQ‐TREE (version 3.0.1) under the best‐fit substitution model Q.INSECT+F+I+R6 with 2,000 ultrafast bootstrap replicates to assess branch support. Both BlastP and tBlastN analyses were performed using *D. melanogaster* Sgf1 and Sgf2 as individual queries to examine the distribution of these genes in each *Drosophila* species.

To determine their evolutionary relationship, we retrieved the Sgf1‐ and Sgf2‐like protein sequences from those species that encode both genes. Sequences were aligned together with Clustal X [68]. The maximum likelihood tree was generated using a Jones‐Taylor‐Thornton model and 500 bootstrap replicates of heuristic searches with the program MEGA 12 [69]. The Sgf2‐like protein (XP_030386857) from the rather basal fly species *S. lebanonensis* (also known as *Drosophila lebanoensis*) was used to root the tree.

### 4.10 Protein Structure Prediction and Comparison

The structures of Sgf1, Sgf2 and their selected homologs from the species *D. simulans* (XP_002076296, Sgf1‐like; XP_016027031, Sgf2‐like), *D. ficusphila* (XP_017038806, Sgf1‐like; XP_017038805, Sgf2‐like), *D. suzukii* (XP_065719383, Sgf2‐like), and *S. lebanonensis* (XP_030386857, Sgf2‐like) were predicted using the algorithm of AlphaFold 3.0 [28]. The C‐terminal region structures of each protein that forms reliable β‐sheets were re‐modeled using PyMol (Ver. 2.5.5, Schrödinger, LLC). Pairwise 3D similarity was aligned by reference to those of Sgf1‐C and Sgf2‐C, respectively, to estimate each TM score using the RCSB.org alignment tool [70].

### 4.11 Protein Deglycosylation Assay

To determine whether the mucin‐like Sgf1 and Sgf2 are glycosylated proteins, we expressed proteins in the Sf9 insect cells using the Bac‐to‐Bac baculovirus expression system [71]. In brief, both genes were individually cloned without their signal peptide regions into the pFastBac vector in fusion with the 6× His tag at the C‐termini. The plasmids were cloned into and amplified in the *E. coli* DH10Bac cells, and the vectors were subsequently transfected into Sf9 cells for incubation for four days at 27°C. The obtained P1 viruses were further used to transfect Sf9 cells to obtain P2 viruses to transfect Sf21 cells for protein expression by growing cells in the Sf‐900 III serum‐free medium (Gibco). After incubation for two days, cells were harvested for protein extraction and purification using the Ni‐NTA Superflow Agarose. The purified proteins were treated with the Protein Deglycosylation Mix II containing the enzyme mixtures, such as O‐glycosidase, β‐N‐acetylhexosaminidase, and PNGase F, and the glycosylated control protein Fetuin (New England Biolabs). Reactions were conducted under both non‐denaturing (native, buffer 1) and denaturing (Buffer 2) conditions for 16 h, respectively. The untreated and treated proteins were subsequently separated by SDS‐PAGE gel analysis and western blotting using the anti‐His antibody to determine the presence/absence or removal of protein smear.

### 4.12 Carbohydrate Polymer and Peptidoglycan Binding Assay

To corroborate the protein binding feature, we performed the binding assays using the β‐1,3 glucan curdlan (isolated from *Alcaligenes faecalis*; Sigma–Aldrich), peptidoglycan (from *Bacillus subtilis*; Sigma–Aldrich), and chitin (purified from shrimp shells; Sigma‐Aldrich) [6]. Protein sample (600 µl each of 0.5 mg/mL for Eac1 and Sgf2, and 1 mg/mL for Sgf1) was added with 10 mg beads in the binding buffer (10 mM Tris‐HCl, pH 7.5, 500 mM NaCl). After incubation at 4°C for 2 h, samples were centrifuged at 12000 rpm for 5 min. The supernatants were carefully collected, and the pellets were washed twice before the addition of 80 µl binding buffer and 20 µL 5× SDS‐PAGE gel loading buffer. The presence or absence of protein in supernatant and bead samples was checked by protein gel analysis after boiling for 5 min.

### 4.13 Fungal and Bacterial Cell Clumping Analysis

Binding of fungal and bacterial cells was also conducted using the purified Sgf1 and Sgf2 proteins with or without the addition of the effector protein Eac1. The conidial spores of *M. robertsii* (WT, Δ*Eac1* and OE::*Eac1*), *B. bassiana*, and *A. fumigatus* were harvested from two‐week‐old PDA plates and suspended in Tris‐buffered saline with Tween‐20 (TBST; 1 M Tris‐HCl, pH = 8.0; 150 mM NaCl; 0.05% Tween 20). Spores were washed twice with TBST buffer and adjusted to a concentration of 1 × 10^8^ conidia/mL. Each cell suspension (100 µL each) was added with 100 µL protein solution (0.5 mg/mL each) plus 400 µL TBST buffer. The same concentration of bovine serum albumin was used as a negative control. The samples were incubated at room temperature for 3 h before photographing in a finger test tube or examining under a microscope (Nikon ECLIPSE Ti2). In addition, the purified Sgf1, Sgf2, and a mixture of both proteins were used to treat Δ*Eac1* spores for imaging by Field‐Emission Scanning Electron Microscopy (Merlin Compact VP, Zeiss) [72]. To disrupt the inter‐ and intra‐protein disulfide bonds, we used the TBST buffer amended with the reducing agents dithiothreitol (DTT; at a final concentration of 10 mM) and β‐mercaptoethanol (β‐ME, 100 mM) to treat Δ*Eac1* spores with the addition of Sgf1 or Sgf2 protein. The hyphal‐body cells of the WT *M. robertsii* harvested above were also used for protein clotting assays.

Bacterial cells of *E. faecalis* and *E. coli* were harvested by centrifugation of their overnight LB cultures. The cells were washed twice with the Tris buffer (1 M, pH = 8.0) and then adjusted to OD600 = 2 for protein binding assays.

### 4.14 Quantitative Analysis of Gene Expressions

To determine the expression of *Eac1*, we extracted RNAs from fungal samples prepared above using the TransZol Up RNA Kit (Transgen Biotech). cDNAs were subsequently synthesized using the HiScript III RT Reagent Kit (Vazyme). RT‐qPCR analysis was conducted using a SYBR mix (Yeasen) and a PikoReal Real‐Time PCR System (Thermo Fisher). The tubulin gene of *M. robertsii* was used as a reference control [5]. RNA samples were also extracted from the female flies of *w^1118^*, *spz^rm7^*, *Tl^KG03609^*, *MyD88^c03881^*, *Dif^1^*, and *Rel^E20^* after topical infection with *M. robertsii* for different durations. Expression of *Sgf1*, *Sgf2*, and the AMP genes for *Drs*, *BaraA*, *Mtk*, *Dso1*, and *BomS1* was performed using different primer pairs (Table S4). The *Drosophila rp49* gene was used as a reference control. Fungal load assays were determined by calibrating the fungal *Rpl32* gene in reference to the *Drosophila Rp49* gene. To this end, 20 flies of each line were collected into a 1.5 mL test tube along with three stainless steel balls (1 mm in diameter) for homogenization at 65 Hz for 1 min, and DNA was extracted as templates using the DNeasy Blood & Tissue Kit (QIAGEN).

### 4.15 Intravital Microscopy Imaging

To determine fungal cell proliferation within the fly body cavity, an IVM imaging strategy was used by following the previous description [73]. First, the WT and mutant female flies were injected with the spores (20 nL each of 1 × 10^8^ conidia/mL) of a GFP‐labeled strain of *M. robertsii* [56]. Flies were anesthetized with diethyl ether instead of CO_2_ 48 h post‐treatment and placed on an oversized cover slip (24 × 60 mm; Beyotime, FCGF60). The fly was positioned with one side of its abdomen facing upward. Confocal images were taken using an inverted Nikon ECLIPSE Ti2 Microscope under default settings. At least five flies of each mutant/treatment were observed, and a representative sample with a common feature was shown.

### 4.16 Quantification and Statistical Analysis

Kaplan–Meier analysis followed by the log‐rank test was conducted to compare insect survival dynamics using GraphPad Prism (ver. 10.1). One‐way ANOVA followed by Tukey's test or two‐tailed Student's *t*‐test was conducted to determine the significance level of difference in gene expression, fungal load, and PO activity between different samples or treatments. Relative gene expression was analyzed using the 2^ΔΔCT^ method [74].

## Author Contributions

C.W. conceived and designed the study, analyzed the data, raised the grants, and wrote the manuscript. S.L. and H.M. performed the experiments and analyzed the data. G.F. performed phylogenetic analysis. D.X., H.W., and C.C. performed library screening and insect bioassays. S.H. performed gene deletion and fungal transformation.

## Conflicts of Interest

The authors declare no conflicts of interest.

## Supporting information




**Supporting File**: advs76623‐sup‐0001‐SuppMat.pdf.

## Data Availability

The data that support the findings of this study are openly available in FlyBase at https://flybase.org/, reference number CG34054, CG30026.
