# Supplementary material for: Subversion of Atypical Mucin Traps by a Spore‐Coat Effector Blocks Cellular Immunity in Drosophila
Source: Adv Sci (Weinh). 2026 Jul 16:e76623. Online ahead of print. doi: 10.1002/advs.76623 (PMC13373891; doi:10.1002/advs.76623)
Supplement: Supplementary file 1 — Supporting File: advs76623‐sup‐0001‐SuppMat.pdf. [file ADVS-9999-e76623-s001.pdf]

Supporting information

Subversion of atypical mucin traps by a spore-coat effector blocks cellular immunity in *Drosophila*

Shiqin Li[1,2]*#, Haimin Chen*[1,2]*#, Gangqi Fang*[1]*, Dongxiang Wei*[1,3]*, Hongyun Wu*[1,2]*, Chen Chen*[1,3]*, Song Hong*[1]*, and Chengshu Wang*[1,2,3]*

[1]Key Laboratory of Insect Developmental and Evolutionary Biology, State Key Laboratory of Plant Trait Design, CAS Center for Excellence in Molecular Plant Sciences, Shanghai Institute of Plant Physiology and Ecology, Chinese Academy of Sciences, Shanghai 200032, China.
[2]School of Life Science and Technology, ShanghaiTech University, Shanghai 201210, China.
[3]CAS Center for Excellence in Biotic Interactions, University of Chinese Academy of Sciences, Beijing 100049, China.

#These authors contributed equally to this study.
*Correspondence: wangcs@sippe.ac.cn

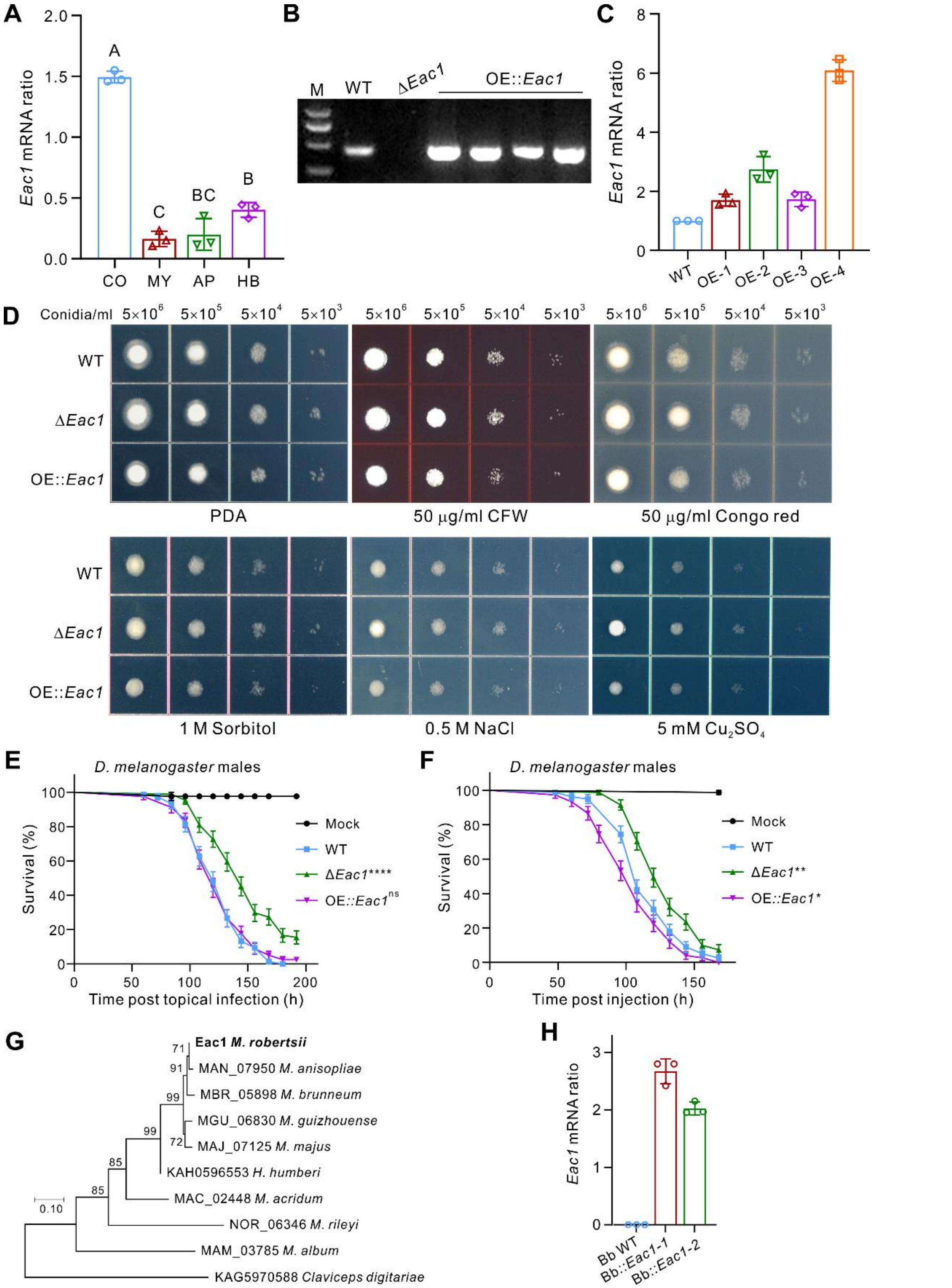

Figure S1. Characterization of *Eac1* gene expression, function, and evolution. A) RT-qPCR analysis of *Eac1* in *M. robertsii* at different developmental stages. CO, conidia harvested from a

two-week-old PDA plate; MY, mycelial cells harvested from the SDB liquid culture three days post-inoculation; AP, appressoria induced on black-soldier fly wings; HB, hyphal-body cells harvested from the hemolymph of wax moth larvae 36 h post-injection. B) PCR and agarose gel electrophoresis analysis to verify *Eac1* gene deletion and overexpression. C) RT-qPCR examination of independent overexpression (OE) mutants. RNA samples were extracted from fungal cultures grown in SDB for 36 h. The independent mutant OE-4 with the highest expression level was used as OE::*Eac1* for further experiments. D) *Eac1* mutant strains have no obvious defects in stress responses. The WT and mutant strains were inoculated (2 μl of each concentration of spore suspensions) on PDA or PDA amended with different compounds for two days. CFW, Calcofluor white. E,F) Differential survival of male flies after topical infection (E) and intra-hemocoel injection (F) with the WT and *Eac1* mutants of *M. robertsii*. Values are the mean ± SEM. Log-rank test between WT and individual mutants: *$p < 0.05$; **$p < 0.01$; ****$p < 0.0001$; ns, not significant. G) Phylogenetic analysis of *Eac1* and its orthologs across *Metarhizium* species. The maximum-likelihood tree was generated with 500 bootstrap replicates. H) RT-qPCR examination of *Eac1* expression in independent overexpression mutants of *B. bassiana*. RNA samples were extracted from fungal cultures grown in SDB for 36 h.

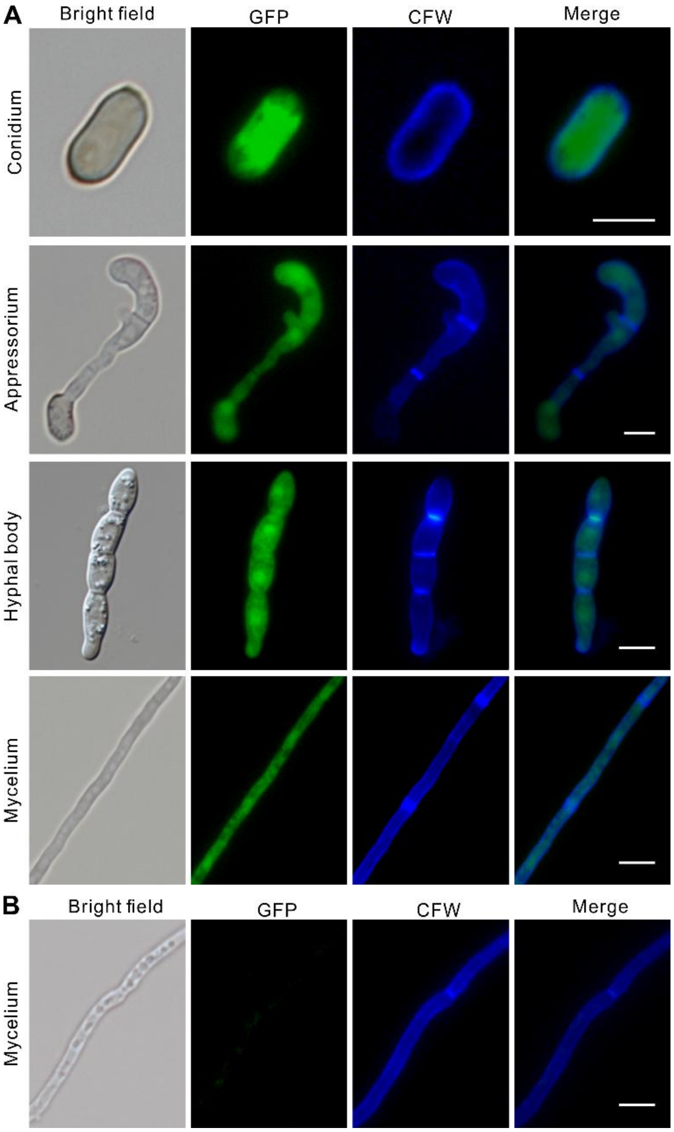

Figure S2. Negative controls of the Eac1 localization assay. A) The GFP-labeled cells of *M. robertsii* show the cytosolic distribution of GFP signal in different cell types. B) The GFP signal was not detected in the mycelium of the Pro_{Eac1}::*SP-GFP-Eac1* strain grown in SDB for 36 h. CFW, Calcofluor White. Bar, 5 μm.

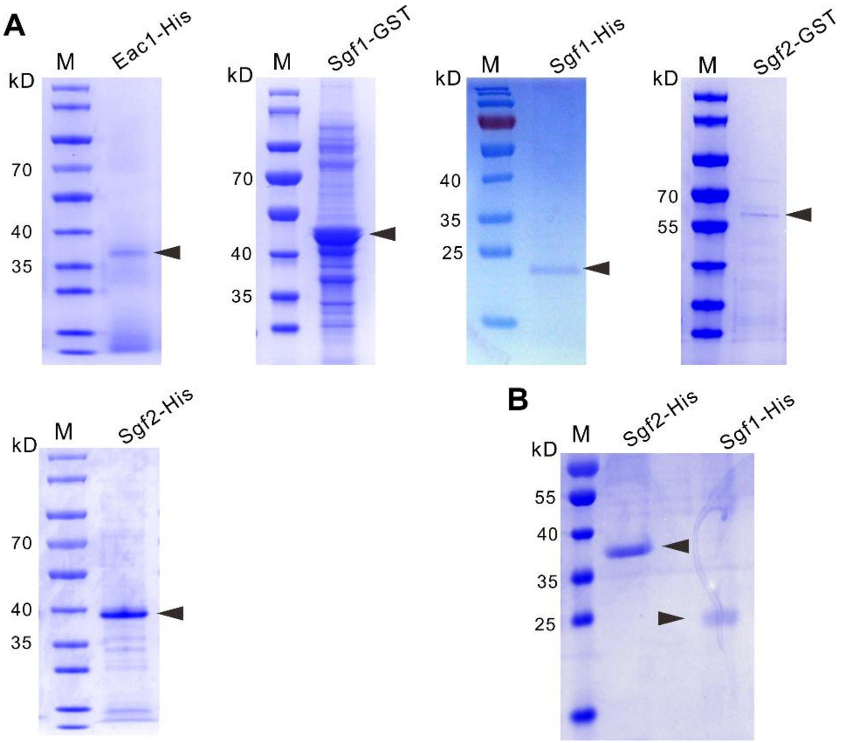

Figure S3. Protein expression and purification assays. A) SDS-PAGE analyses show proteins expressed by *E. coli* cells. B) SDS-PAGE analysis shows proteins expressed in Sf9 cells. Proteins were fused with different tags for expression and purification. Target proteins are arrowed.

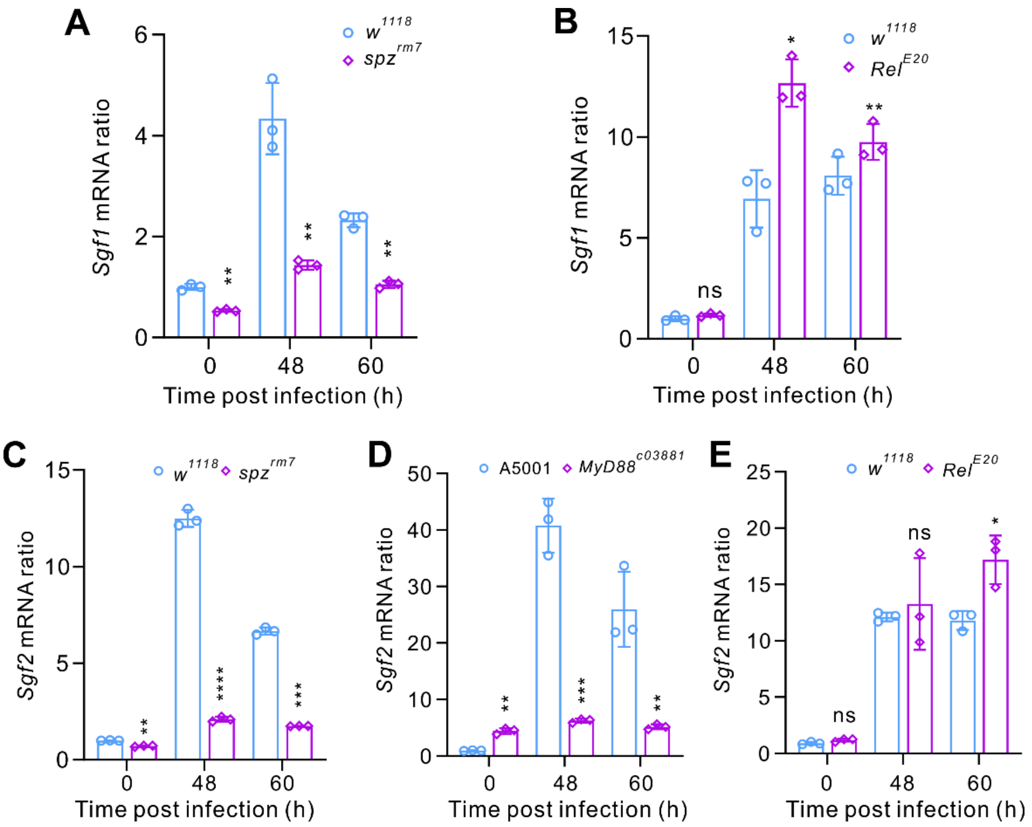

Figure S4. RT-qPCR analysis of gene transcription. A,B) Reduced expression of *Sgf1* in *spz*^{rm7} (A) but not *Rel*^{E20} (B) female flies after topical infection with the WT *M. robertsii*. C-E) Reduced expression of *Sgf2* in *spz*^{rm7} (C), *MyD88*^{c03881} (D), but not *Rel*^{E20} (E) female flies after topical fungal infection with the WT *M. robertsii*. Pairwise two-tailed Student's *t*-test: *$p < 0.05$; **$p < 0.01$; ***$p < 0.001$; ****$p < 0.0001$; ns, not significant.

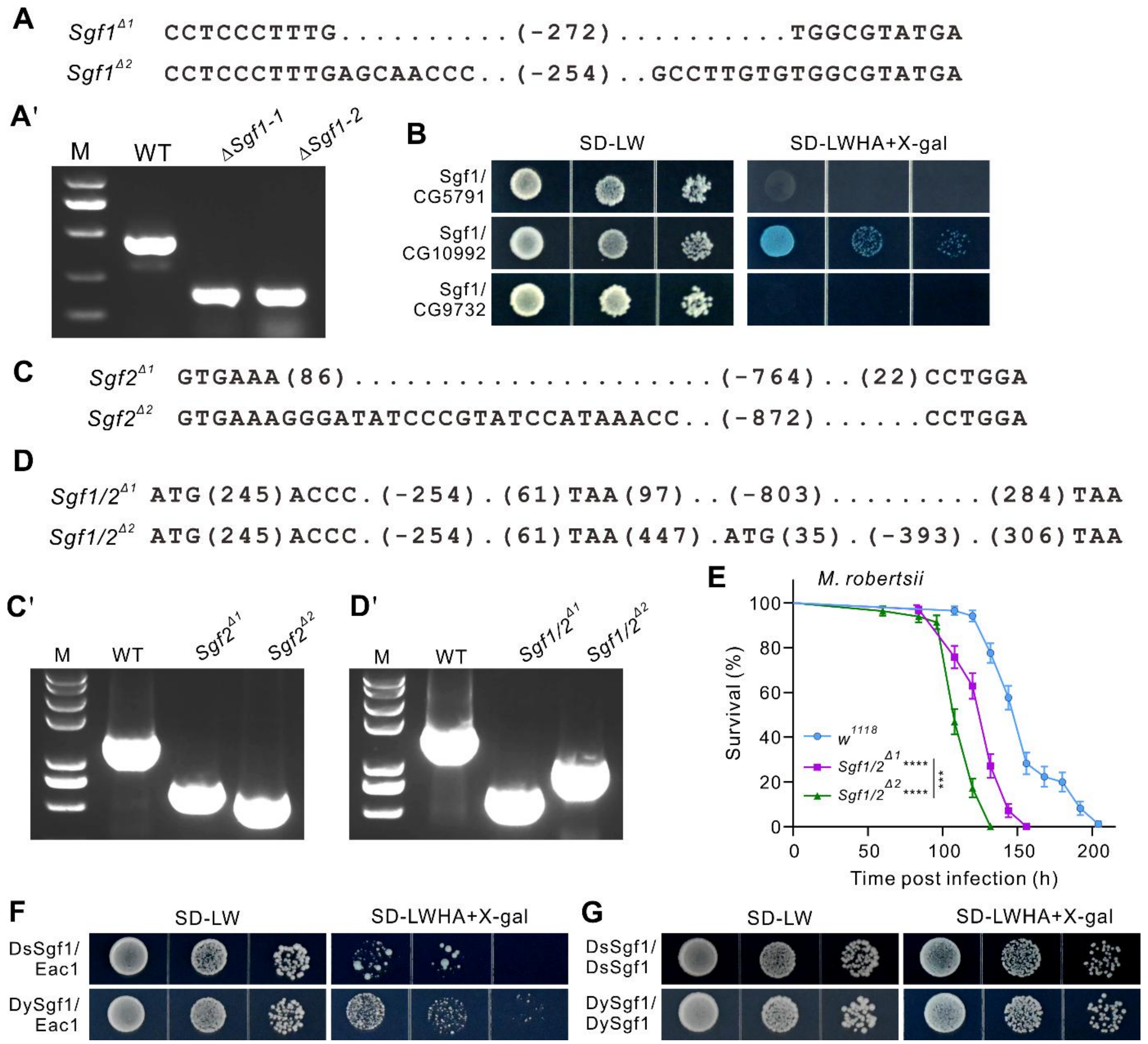

Figure S5. Verification of gene disruptions in *Drosophila* and survival assays. A) PCR sequencing verifies independent disruptions of *Sgf1*. B) Y2H analysis verifies putative targets that interact with Sgf1 or not. C,D) PCR sequencing verifies independent disruptions of *Sgf2* (C) and *Sgf1/2* double genes (D). Panels A′, C′, and D′ show the corresponding PCR product bands for A, C, and D. E) Double deletion of *Sgf1* and *Sgf2* significantly increased the susceptibility of female flies to the topical infection of WT *M. robertsii*. Values are the mean ± SEM; Log-rank test: ***$p < 0.001$; ****$p < 0.0001$. F,G) Y2H analysis confirms the positive interactions between Eac1 and DsSgf1/DySgf1 (F), and the self-interactions of DsSgf1 and DySgf1. DsSgf1, Sgf1-like homolog from *D. simulans* (XP_002076296); DySgf1, Sgf1-like homolog from *D. yakuba* (XP_043062837).

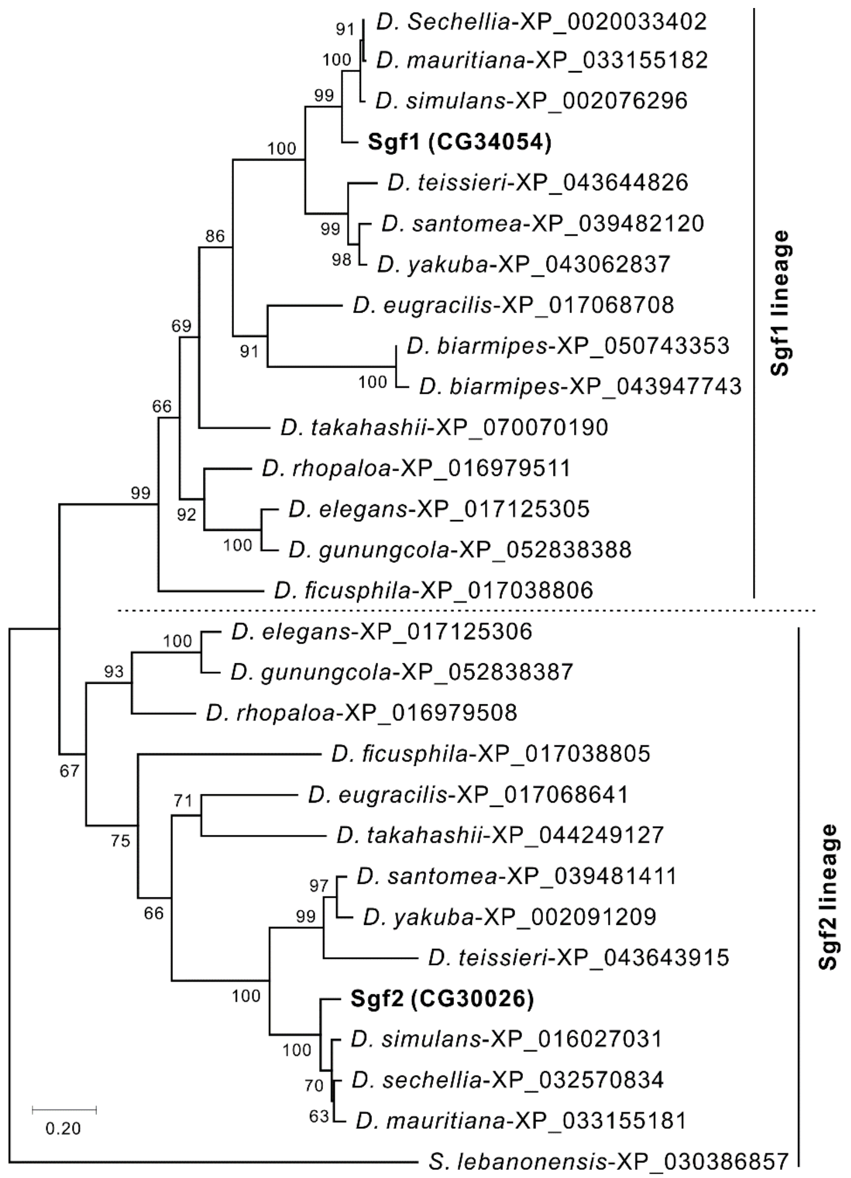

Figure S6. Phylogenetic analysis of Sgf1/Sgf2 and their homologs encoded by the selected *Drosophila* species. The maximum likelihood tree was generated using a Jones-Taylor-Thornton model and 500 bootstrap replicates of heuristic searches. The tree with the highest log likelihood (-7,294.90) is shown here. The Sgf2-like protein from the rather basal fly *Scaptodrosophila lebanonensis* (also known as *Drosophila lebanoensis*) is used to root the tree.

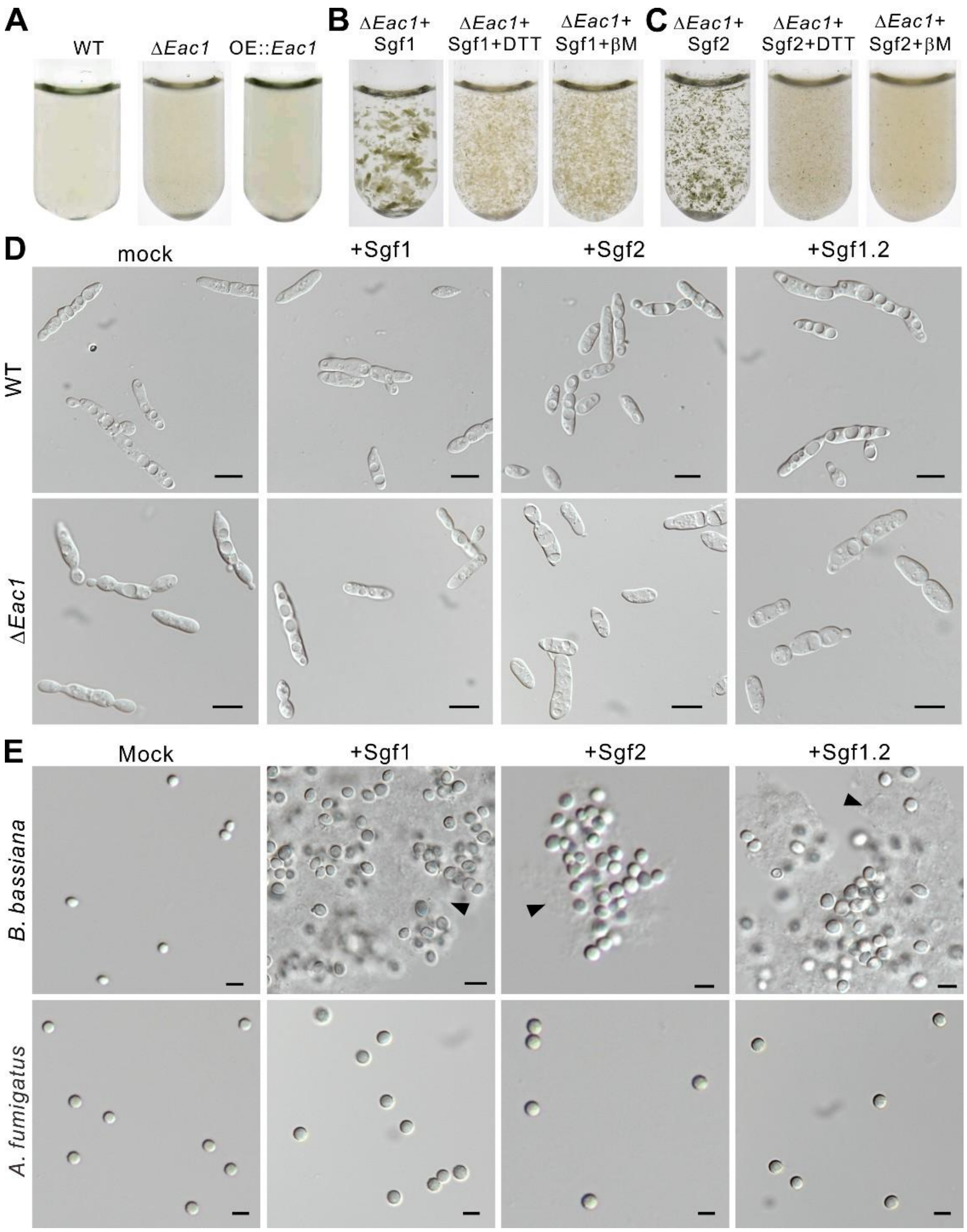

Figure S7. Clumping or non-clumping of fungal cells by different proteins. A) Spore suspensions of the WT and *Eac1* mutants without the addition of any protein. The conidia of each strain were suspended in 0.05% Tween 20 at the same concentration of 5×10^7 conidia/ml. B,C) Reducing agents inactivate the clotting activities of Sgf1 (B) and Sgf2 (C) against Δ*Eac1* spores in glass tube assays. DTT, dithiothreitol (10 mM); βM, β-mercaptoethanol (100 mM). D) Microscopic images show that WT and Δ*Eac1* hyphal-body cells are not entrapped by Sgf1 and Sgf2 either individually or in combination. E) Microscopic images show the clumping or non-clumping of *B. bassiana* and *A. fumigatus* conidial spores by Sgf1 and Sgf2. Bar, 5 μm.

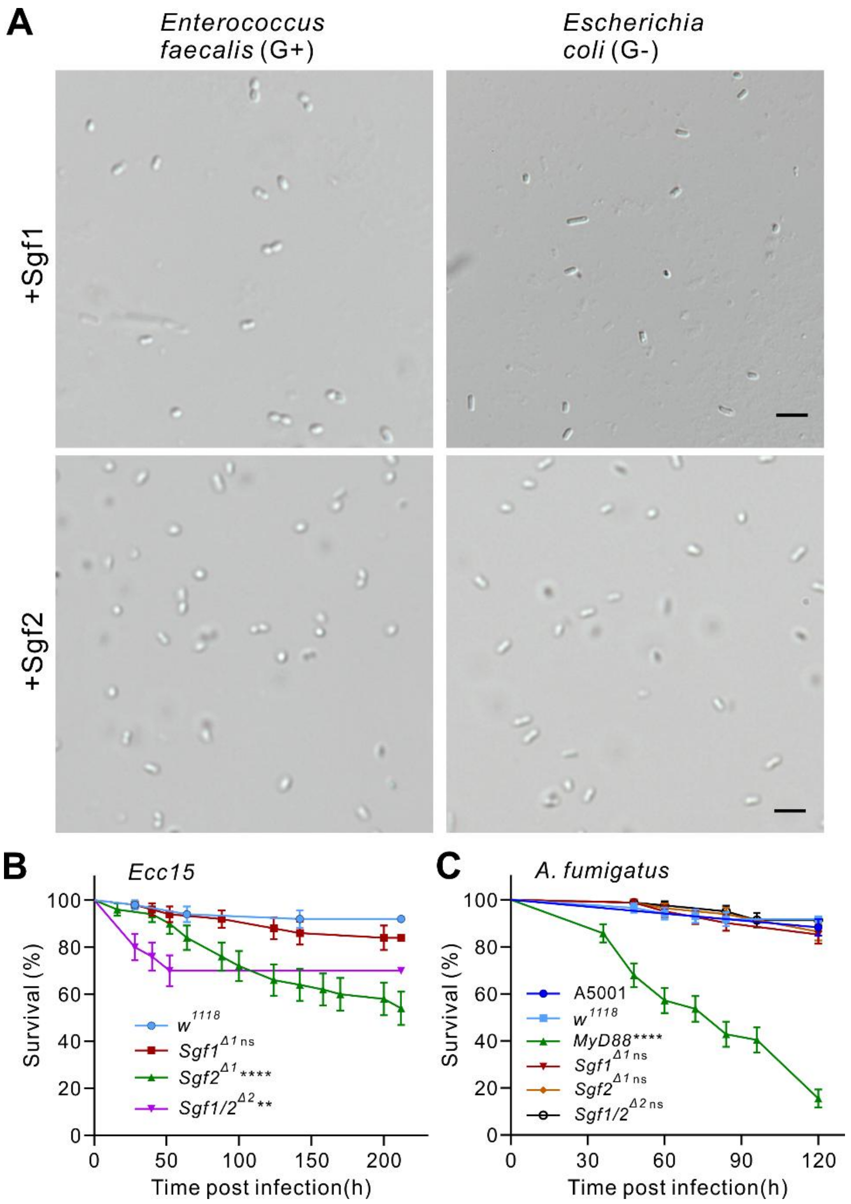

Figure S8. Bacterial cell clumping and survival assays. A) Non-clotting of different G+ and G- bacterial cells by Sgf1 and Sgf2. Bar, 2 µm. B) Disruption of *Sgf1*, *Sgf2* or *Sgf1/2* in *Drosophila* differentially affected female fly survival after injection with the *Ecc15* cells. Each insect was injected with 25 nl of bacterial cells (OD600 = 6). C) Disruption of *Sgf1*, *Sgf2* or *Sgf1/2* in *Drosophila* did not affect female fly survival after injection with the spores of *A. fumigatus* (10 nl each of 5×10^8 conidia/ml). $MyD88^{c03881}$ flies were included as a negative control. Plotted values are the mean ± SEM. Log-rank test: ns, not significant; $**p < 0.01$; $****p < 0.0001$.

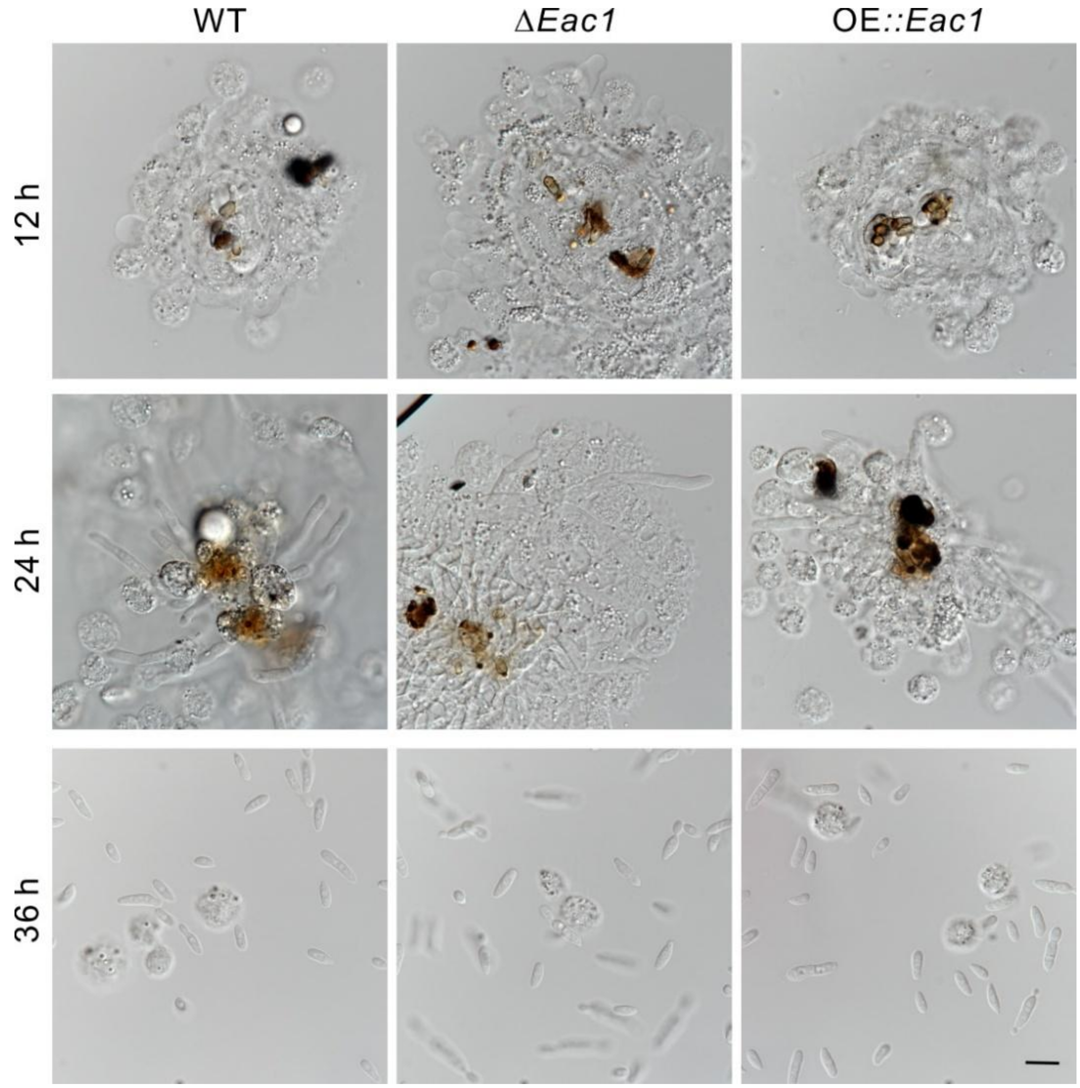

Figure S9. Similar evasion of the cellular immune defenses of wax moth larvae after injection with the WT and *Eac1* mutant strains of *M. robertsii*. The last instar larvae were individually injected with 10 µl of spore suspension (2 × 10^7 conidia/ml), and the insects were bled for microscopic imaging after different durations as indicated. The injected conidial spores of the WT and mutant strains were similarly encapsulated and melanized by hemocytes 12 h post-injection. Fungal cells escaped from the nodules 24 h post-injection, and a similar number of hyphal-body cells was formed by WT and mutant strains 36 h post-injection. Bar, 5 µm.

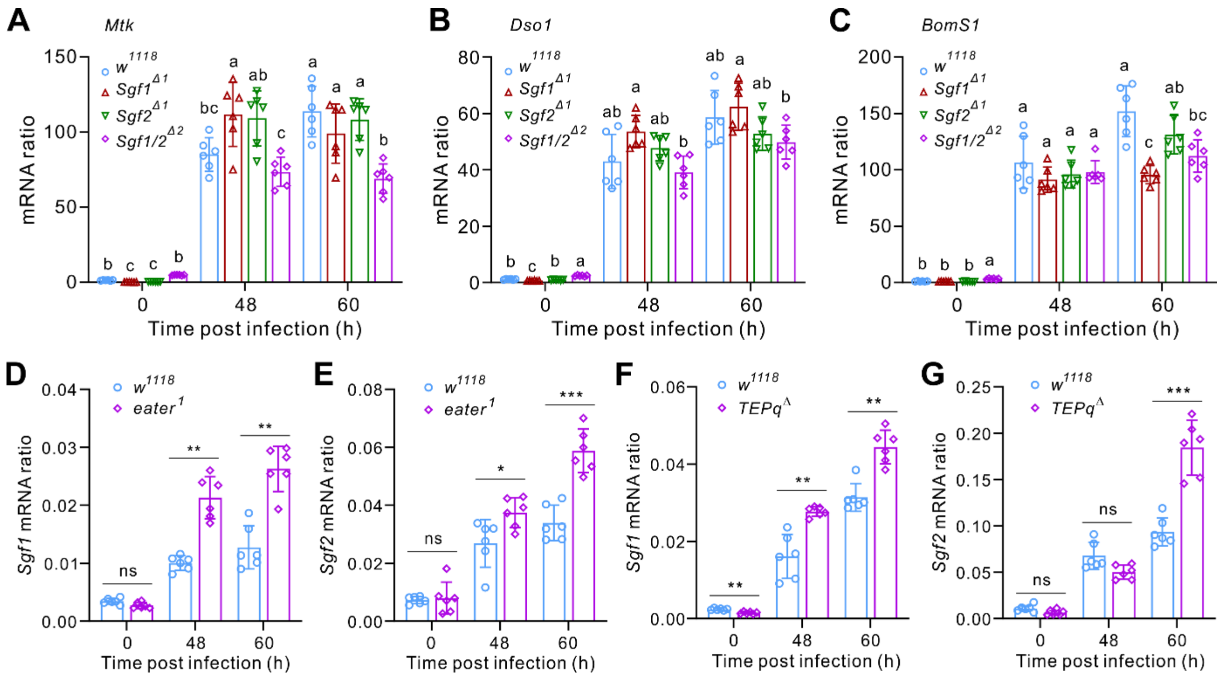

Figure S10. Differential gene expression in different *Drosophila* lines after topical infection with the WT *M. robertsii*. A-C) Differential expression of the antifungal genes *Mtk* (A), *Dso1* (B), and *BomS1* (C) in *Sgf1* and *Sgf2* mutant females after topical infection with the WT *M. robertsii* for different durations. D,E) Upregulation of *Sgf1* (D) and *Sgf2* (E) in *eater*^{1} female flies after topical fungal infection. F,G) Upregulation of *Sgf1* (F) and *Sgf2* (G) in *TEPq*$^{\Delta}$ female flies after topical fungal infection. Values are the mean ± SD. Panels A-C: One-way ANOVA followed by Tukey's tests among samples: different letters labeled above column, $p < 0.05$. Panels D-H: Pairwise two-tailed Student's *t*-test: *$p < 0.05$; **$p < 0.01$; ***$p < 0.001$; ns, not significant.

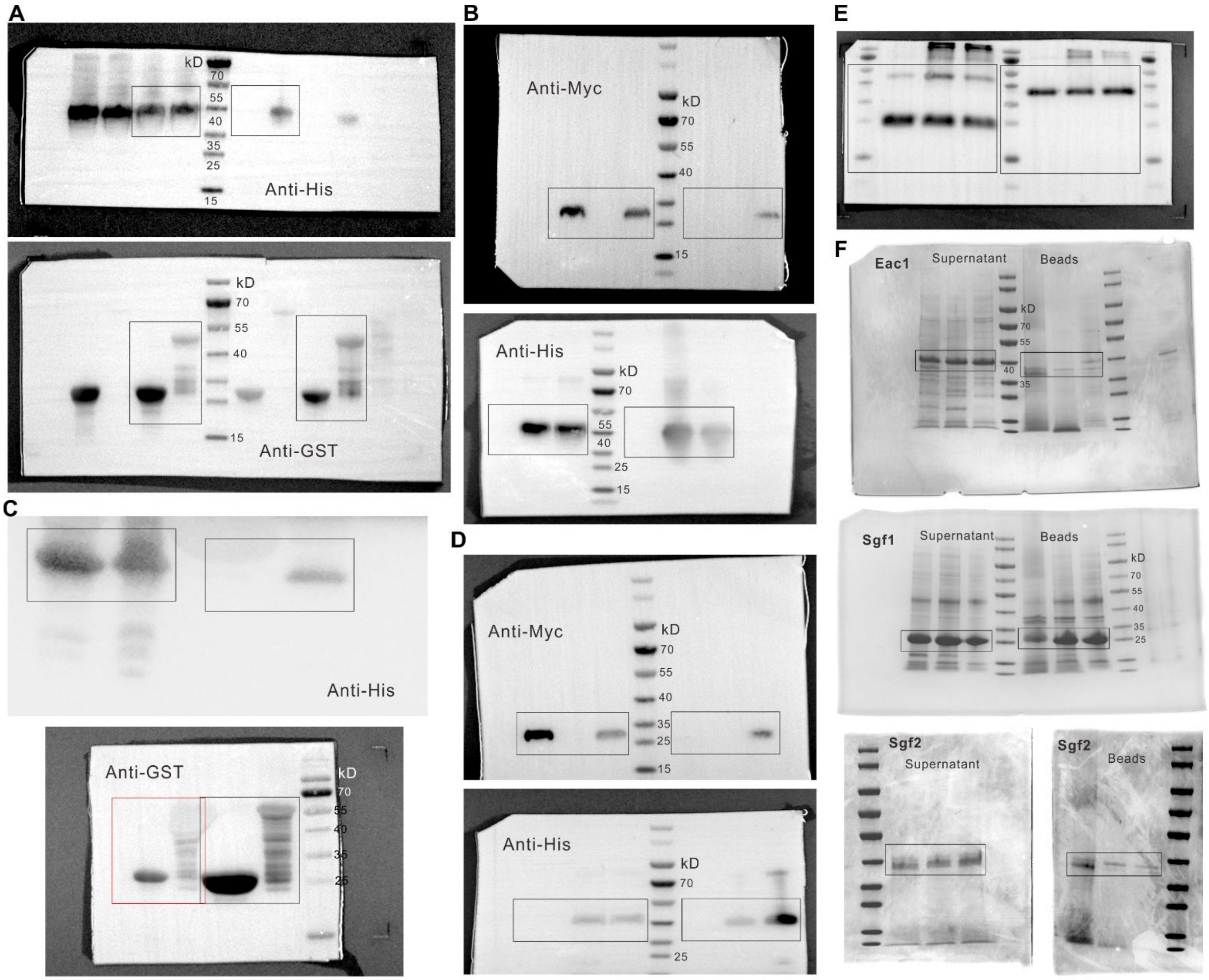

Figure S11. Original images used for generating different figure panels in the main context. A,B) Original images for generating Figure 2B (A) and 2C (B). C,D) Original images for generating Figure 3B (A) and 3C (B). E) Original images for generating Figure 4G. F) Original images for generating Figure 5B. The corresponding regions used in the main figures are framed.

Table S1. Library screening hits using Eac1 as bait.

Clone no.	Flybase gene	Signal peptide (aa)	Annotation
1	CG3481	No	alcohol dehydrogenase, isoform C
2	CG4094	No	fumarase 1, transcript variant B (Fum1), mRNA
1	CG18067	1-22	Bombardier
1	CG8882	No	eukaryotic translation initiation factor 3 subunit I
5	CG34054	1-21	uncharacterized protein
1	CG44243	No	lipoyltransferase 1, isoform B

Table S2. Library screening hits using Sgf1 as bait.

Clone no.	Flybase gene	Signal peptide (aa)	Definition
5	CG12116	No	uncharacterized protein
5	CG5791	1-18	Bomanin bicipital 3, isoform B
9	CG10992	1-19	Cathepsin B1, isoform C
3	CG30026	1-17	uncharacterized protein
12	CG18745	No	uncharacterized protein, arrestin family protein
1	CG9372	1-21	uncharacterized protein
2	CG2238	No	eukaryotic translation elongation factor 2, isoform A
1	CG17691	No	2-oxoisovalerate dehydrogenase subunit beta
2	CG9882	No	arginine methyltransferase 7
1	CG5214	No	uncharacterized protein
2	CG5380	No	RNA polymerase III subunit F
1	CG8588	No	Pastrel, isoform G
1	CG12548	No	No mechanoreceptor potential B, isoform C
1	CG11981	No	proteasome beta3 subunit
1	CG8386	No	Ubiquitin-fold modifier conjugating enzyme 1

Table S3. Materials and reagents used in this study.

Reagent or resource	Source/Manufacturer	Identifier
**Bacterial and fungal strains**		
Metarhizium robertsii	USDA	ARSEF 23
M. robertsii ΔEac1	This study	N/A
M. robertsii OE::Eac1	This study	N/A
Beauveria bassiana	USDA	ARSEF 2860
B. bassiana OE::Eac1	This study	N/A
Aspergillus fumigatus Af293	FGSC	A1100
Saccharomyces cerevisiae	Weidi Biotech	Strain# Y2H Gold
Saccharomyces cerevisiae	Weidi Biotech	Strain# AH109
Escherichia coli	Weidi Biotech	Strain# Top10
Escherichia coli	Weidi Biotech	Strain# Rosetta
Agrobacterium tumefaciens	Weidi Biotech	Strain# AGL1
Enterococcus faecalis	Ref. 52	N/A
Erwinia carotovora carotovora 15	Ref. 52	N/A
Drosophila **stocks**		
Drosophila melanogaster w^{1118}	BDSC	#5905
D. melanogaster A5001	Ref. 9	N/A
D. melanogaster yw	Ref. 9.	N/A
D. melanogaster $MyD88^{c03881}$	Ref. 9	N/A
D. melanogaster $Tl^{KG03609}$	BDSC	13022
D. melanogaster Dif^{1}	Ref. 9	N/A
D. melanogaster $eater^{1}$	Ref. 17	N/A
D. melanogaster $TEPq^{\Delta}$	Ref. 18	N/A
D. melanogaster $w;Ubi\text{-}Gal4;tub\text{-}Gal80^{ts}$	Guangzhou Medical University	N/A
D. melanogaster $w;Da\text{-}Gal4;Tub\text{-}Gal80^{ts}$	Guangzhou Medical University	N/A
D. melanogaster UAS-mCherry	Ref. 6	N/A
D. melanogaster $Sgf1^{\Delta}$	This study	N/A
D. melanogaster $Sgf2^{\Delta}$	This study	N/A
D. melanogaster $Sgf1/2^{\Delta}$	This study	N/A
D. melanogaster UAS-*Sgf1*	This study	N/A
D. melanogaster UAS-*Sgf2*	This study	N/A
D. simulans	NDRCC	BCF #93
D. yakuba	NDRCC	BCF #94
D. virilis	NDRCC	BCF #97
D. suzukii	Ref. 9	N/A
**Antibodies**		
Mouse anti-GST antibody	Proteintech	Cat# 66001-2-Ig; WB (1:5000)
Mouse anti-6× His antibody	Proteintech	Cat# 66005-1-Ig; WB (1:10000)
Myc-Tag Rabbit mAb	ABclonal	Cat# AE070; WB

		(1:10000)
**Media and chemicals**		
Potato dextrose agar	BD Difco	Cat# 213330
Sabouraud dextrose broth	BD Difco	Cat# 238230
Sf-900 III serum-free medium	Gibco	Cat# 12658019
Aureobasidin A	YEASEN	Cat# 60231ES08
Chlorimuron ethyl	YEASEN	Cat# 41015ES60
Glufosinate ammonium	Sigma-Aldrich	Cat# 45520
Chitin	Sigma-Aldrich	Cat# C9752
Peptidoglycan	Sigma-Aldrich	Cat# 69554
Curdlan	Sigma-Aldrich	Cat# G5011
Calcofluor White	Sigma-Aldrich	Cat# 18909
SD-Leu/-Trp	TaKaRa	Cat# 630417
SD-Ade/-His/-Leu/-Trp	TaKaRa	Cat# 630428
SD-His/-Leu/-Met/-Trp	TaKaRa	Cat# 630429
X-Gal	YEASEN	Cat# 10901ES03
L-DOPA	SparkJade	Cat# SJ-MN0431
Ni-NTA Superflow Agarose	Thermo Fisher	Cat# 25217
TRIzol reagent	Thermo Fisher	Cat# 15596026
**Commercial kits**		
2 × Phanta Max Master Mix (Dye Plus)	Vazyme	Cat# P525-01
HiScript III RT SuperMix for qPCR (+gDNA wiper)	Vazyme	Cat# R323-01
Taq Pro Universal SYBR qPCR Master Mix	Vazyme	Cat# Q712-02
DNeasy Blood & Tissue Kit	Qiagen	Cat# 69504
TransZol Up RNA kit	TransGen Biotech	Cat# ER501-01
Myc-tag Protein IP Assay Kit with Magnetic Beads	Beyotime	Cat# P2183S
PikoReal Real-Time PCR System	Thermo Fisher	Cat# N11471
Protein Deglycosylation Mix II	New England Biolabs	Cat# P6044S
**Recombinant DNA**		
pDHt-Bar	Ref. 59	N/A
pDHt-Sur	Ref. 59	N/A
pGBKT7	Takara Bio	Cat# 630443
pGADT7	Takara Bio	Cat# 630442
pGex-6p-1	Addgene	Cat# 38315
pET-28b	Addgene	Cat# 69865–3
pUAST-attB	Drosophila Genomics Resource Center	DGRC_1419

Table S4. PCR primers used in this study.

Primers	Primer sequence	Note
**Fungal genetic transformation**		
Eac1OE-F	ATCGAATTCCTGCAGATGAAGGCCTCTCAAGTTCT	Gene
Eac1OE-R	TCCCCCGGGCTGCAGTCACAGCATCATGGCG	overexpression
BbEac1OE-F	TCAATAACAACTAGTTCTAGAATGAAGGCCTCTCAAGTT	Overexpression in
BbEac1OE-R	CGCGGTGGCGGCCGCTCTAGATCACAGCATCATGGCGGC	*B. bassiana*
sur-proSP-F	ATCGAATTCCTGCAGATGTTGAGCGGCTCCAGTTC	Cloning self-promoter
EGFP-SP-R	CTCGCCCTTGCTCACCATAGCCAGGGCAGGGGCGAC	
SP-EGFP-F	GTCGCCCCTGCCCTGGCTATGGTGAGCAAGGGCGAG	
Eac1-EGFP-R	AATCTCGGTGCGGTTCTGCTTGTACAGCTCGTCCAT	
M13F	GTAAAACGACGGCCAGT	Plasmid sequencing
M13R	CAGGAAACAGCTATGAC	
**Interaction analysis analysis**		
Eac1BK-F	GAGGAGGACCTGCATATGCAGAACCGCACCGAGATTGT	
Eac1BK-R	CGCTGCAGGTCGACGGATCCTCACAGCATCATGGCG	
Sgf1BK-F	GAGGAGGACCTGCATATGCAGGATGTTATACCAGAG	
Sgf1BK-R	CGCTGCAGGTCGACGGATCCTTACAATACTTGTTTCCAAT	
Sgf2BK-F	GAGGAGGACCTGCATATGACTTCTTTATCCGCCGAACC	
Sgf2BK-R	CGCTGCAGGTCGACGGATCCTTACTTGGTCGGCCTCTGCT	
Sgf1AD-F	CCAGATTACGCTCATATGCAGGATGTTATACCAGAG	
Sgf1AD-R	CTCGAGCTCGATGGATCCTCATTACAATACTTGTTTCCAAT	
Sgf1C-AD-F	CCAGATTACGCTCATATGGTGTGGATGCAACCGATA	
Sgf1C-AD-R	CTCGAGCTCGATGGATCCTCACAATACTTGTTTCCAATTTA	
Sgf1C-BK-F	GAGGAGGACCTGCATATGGTGTGGATGCAACCGATA	
Sgf1C-BK-R	CGCTGCAGGTCGACGGATCCCAATACTTGTTTCCAATT	
Sgf2AD-F	CCAGATTACGCTCATATGCAGGATACAAGGCCAGAAGT	
Sgf2AD-R	CTCGAGCTCGATGGATCCTCATTAATAGACATAGAAATTCGG	
Sgf2C-AD-F	CCAGATTACGCTCATATGGTAAAATGTTATCTGCGAAA	Y2H analysis
Sgf2C-AD-R	CTCGAGCTCGATGGATCCTCAATAGACATAGAAATTCGGTG	
Sgf2C-BK-F	GAGGAGGACCTGCATATGGTAAAATGTTATCTGCGAAA	
Sgf2C-BK-R	CGCTGCAGGTCGACGGATCCATAGACATAGAAATTCGGTG	
CG10992AD-F	CCAGATTACGCTCATATGGAGCCGTCACTGCTCTCG	
CG10992AD-R	CTCGAGCTCGATGGATCCTCATTACAGCTTGGGCAGACC	
CG3481AD-F	CCAGATTACGCTCATATGATGTCGTTTACTTTGACCA	
CG3481AD-R	CTCGAGCTCGATGGATCCTCATTAGATGCCGGAGTCCCAGT	
CG8882AD-F	CCAGATTACGCTCATATGATGCGTCCGTTGATGCTTCA	
CG8882AD-R	CTCGAGCTCGATGGATCCTCACTACTCGAAGATGTTCTCA	
CG4094AD-F	CCAGATTACGCTCATATGATGGCCTCCCAGGAGTTTC	
CG4094AD-R	CTCGAGCTCGATGGATCCTCATCACTTGGGTCCCAGCATC	
CG44243AD-F	CCAGATTACGCTCATATGATGAGCATGCTGTTGCGAC	
CG44243AD-R	CTCGAGCTCGATGGATCCTCATCAAGCTGATCCGTTTATTG	
DsSgf1-AD-F	CCAGATTACGCTCATATGCAGGATGTTATACCAGAG	
DsSgf1-AD-R	CTCGAGCTCGATGGATCCTCACAATACTTGTTTCCAATT	
DsSgf1-BK-F	GAGGAGGACCTGCATATGCAGGATGTTATACCAGAG	
DsSgf1-BK-R	CGCTGCAGGTCGACGGATCCCAATACTTGTTTCCAATT	
DySgf1-AD-F	CCAGATTACGCTCATATGCAGGATATTATTCCAGAGATACCAGA	
DySgf1-AD-R	CTCGAGCTCGATGGATCCTCACAATACTTCTTTCCAATTTACGTGTA	
DySgf1-BK-F	GAGGAGGACCTGCATATGCAGGATATTATTCCAGAGATACCAGA	
DySgf1-BK-R	CGCTGCAGGTCGACGGATCCCAATACTTCTTTCCAATTTACGTGTA	
T7	TAATACGACTCACTATAGGGCGAGCG	pGADT7 insert
3-AD	GTGAACTTGCGGGGTTTTTCAGTAT	sequencing
5BD	GTGCGACATCATCATCGGAAG	pGBKT7insert
3BD	TAAGAGTCACTTTAAAATTTGTATAC	sequencing
1st-15293-F	GAATTCCCGGGGATCCGTACTTCTTTATCCGCCGAACC	
1st-15293-R	TGCAGGTCGACGGATCCCTTACTTGGTCGGCCTCTGCT	
1st-Sgf1-F	GAATTCCCGGGGATCCGTCAGGATGTTATACCAGAG	Y3H analysis
1st-Sgf1-R	TGCAGGTCGACGGATCCCTTACAATACTTGTTTCCAAT	
2nd-Eac1-F	AGAAAGGTGGCGGCCGCACAGAACCGCACCGAGATTGTC	

2nd-Eac1-R	TCGGGCTAATGCGGCCGCTCACAGCATCATGGCGGC	
MET17-F	CTATTACCCCCATCCATACA	
MET17-R	ACACCTGGCAATTCCTTACC	
**Protein expression in *E. coli***		
Eac1-28bF	AGAAGGAGATATACCATGGGCATGCAGAACCGCACCGAGATTGT	
Eac1-28bR	GTGGTGGTGCTCGAGCAGCATCATGGCGGC	
Sgf1-28bF	AGAAGGAGATATACCATGGGCCAGGATGTTATACCAGAGAT	
Sgf1-28bR	GTGGTGGTGCTCGAGCAATACTTGTTTCCAATT	
Sgf2-28bF	AGAAGGAGATATACCATGGGCATGCAGGATACAAGGCCAGAAGT	
Sgf2-28bR	GTGGTGGTGCTCGAGATAGACATAGAAATTCGG	
Sgf1GST-F	CAGGGGCCCCTGGGATCCATGCAGGATGTTATACCAGAGAT	
Sgf1GST-R	TCGACCCGGGAATTCCGGTTACAATACTTGTTTCCA	
Sgf2GST-F	CAGGGGCCCCTGGGATCCATGCAGGATACAAGGCCAGAAGT	
Sgf2GST-R	TCGACCCGGGAATTCCGGTTAATAGACATAGAAATTCGG	
***Drosophila* transgenesis**		
UAS-Sgf1F	AGATCTGCGGCCGCGGCTCGAGATGTATTTTGGCAAAGTA	
UAS-Sgf1V5R	CCTTCACAAAGATCCTCTAGATTACGTAGAATCGAGACCGAGGAGAG<br>GGTTAGGGATAGGCTTACCCAATACTTGTTTCCA	
UAS-Sgf2F	AGATCTGCGGCCGCGGCTCGAGATGAAGTCTGCTATTATTTG	
UAS-Sgf2V5R	CCTTCACAAAGATCCTCTAGATTACGTAGAATCGAGACCGAGGAGAG<br>GGTTAGGGATAGGCTTACCATAGACATAGAAATTCG	
**Deletion and verification of gene deletion in *Drosophila***		
Sgf1Sg1	CTCCCTTTGAGCAACCCACA	
Sgf1g1Sg2	CATACGCCACACAAGGCCCT	
Sgf2Sg1	GGAGGTATCCCTGAGGTCGA	Guard RNAs for gene deletion
Sgf2Sg3	CTTCATCGCGACTGCAACGT	
DKOSg1	TGCTCAACTTCTATAATAAC	
Sgf1KO-IF	AAAATCCACCACAACTACAA	
Sgf1KO-IR	CTGTTCCTGCTTAATTTCTT	
Sgf2KO-IF	AGATTCCTGGGGTGTGGATG	Verification of gene deletion
DoubleKO-IF	GCTTCCGTTAGCACCGTTTG	
DoubleKO-IR	ATCGTACCACGAACACCGTC	
**RT-qPCR**		
TubQF1	GTCACCACATGCTTGCGTTT	
TubQR1	GTCGAACATCTGCTGGGTGA	
Eac1-F	TCCTGTGCCTACCACTATTC	
Eac1-R	CATTGTTACCGCCGTCGTTG	
RP49-qF	GCCCAAGGGTATCGACAACA	
RP49-qR	CTTGCGCTTCTTGGAGGAGA	
Sgf1-qF	CACACGGACAACCGAGATTC	
Sgf1-qR	AGCAGCAGCGATAACAAGTG	
Sgf2qF	TATTTGCGTGTTCTTCATC	
Sgf2qR	GTCGTTACTGGTGCCTCT	
Sgf3qPCR-F	TAAATGACCGAAGACTGGAC	
Sgf3qPCR-R	CATTTATCCAAAGCATCGTG	
Daisho2-qF	ATGAACTGTCTGAAGATCTGCGGC	
Daisho2-qR	GCGTATATACTGCGAGCGATCAG	
Daisho1-qF	CTTCTCTTGGCCATGTTCGCT	
Daisho1-qR	ATGTACTGGGTGTTGTCGGTC	
Metch-qF	GCAACTTAATCTTGGAGCGA	
Metch-qR	CGGTCTTGGTTGGTTAGGAT	
Drs-qF	GTACTTGTTCGCCCTCTTCG	
Drs-qR	CTCCTCCTTGCACACACGAC	
BaraA2-qF	CCACAACACACCTACGACGG	
BaraA2-qR	CGGAAAAATTGGGACCACTG	
BomS1-qF	CACCGTTTTTGTGCTCGGTC	
BomS1-qR	CGTGGACATTGCACACCCT	
BomBc2-qF	GACTGGGCACTGCATCAATC	
BomBc2-qR	GCAACCGACGCAATCACTAT	
Spz1-qF	CCTTTGCAGGAGCATCAGGA	
Spz1-qR	GGTCTGCTGTGTGTAGTGCT	

Rpl32-F	ATCGGTTTCGGGTCCAAC	*Metarhizium Rpl32*
Rpl32-R	ATTCGCTTTCGGGAGGAG	
Act-F	GCCAACCGTGAGAAGATGA	*Drosophila Actin 5C*
Act-R	GGTGGTGAAAGAGTAACCGC	
